# Medium-adaptive wideband near-field antenna for microwave detection of internal cavities in plant stems

**DOI:** 10.1038/s41598-026-61146-w

**Published:** 2026-07-13

**Authors:** Mariam M. Elkial, Khalid. F. A. Hussein, Manal Mustafa

**Affiliations:** 1 Electrical Engineering Department, Al-Azhar University, Cairo, Egypt; 2 Microwave Engineering Department, Electronics Research Institute (ERI), Cairo, Egypt; 3 Computer Engineering Department, Al-Azhar University, Cairo, Egypt

**Keywords:** Near-field antenna, Wideband antenna, Medium-adaptive antenna, Microwave sensing, Non-destructive evaluation (NDE), Mutual coupling, Plant stem inspection, Tree health monitoring, Balun design, Microwave radar sensing, Engineering, Physics

## Abstract

This paper presents a novel medium-adaptive wideband near-field antenna for early detection of internal cavities in plant stems, including branches and small trunks. Unlike conventional antennas designed for free-space operation, the proposed antenna is explicitly engineered to operate in close proximity to a lossy, anisotropic, and dispersive cylindrical medium representing wood tissues. A physics-based electromagnetic model of the stem is incorporated into the design process, enabling accurate optimization under realistic dielectric loading conditions. The antenna consists of a compact quasi-planar dipole with blended arms integrated with a medium-adaptive dual-ring balun that ensures balanced current excitation and stable impedance matching under strong near-field loading. Both simulation and experimental measurements demonstrate wideband impedance matching to a 50 Ω source over the 2.0–3.0 GHz frequency range. Surface current distribution and specific absorption rate (SAR) analyses confirm efficient electromagnetic coupling into the stem tissues with minimal radiation leakage. To evaluate the sensing capability of the proposed design, a conceptual two-element antenna system is introduced as a feasibility study for cavity detection. The detection performance is assessed through a sensitivity-driven framework based on variations in both self- and mutual-scattering parameters. A comprehensive sensitivity analysis is conducted to quantify the response of the system to changes in cavity diameter, radial position, and angular location. The results demonstrate that while the reflection coefficient is primarily sensitive to near-surface inhomogeneities, the mutual coupling between antenna elements provides strong and reliable sensitivity to internal cavity characteristics. Based on the sensitivity analysis, an optimal operating frequency band centered at 2.76 GHz and an appropriate antenna clearance are identified to maximize detection performance. The proposed antenna and sensing methodology are further validated through experimental measurements, confirming the consistency with numerical results. Simulation results demonstrate cavity-detection sensitivity, while experimental measurements validate the antenna impedance matching and mutual-coupling characteristics. The compact geometry of the antenna enables scalable multi-element configurations, establishing a practical framework for non-destructive, microwave-based monitoring of internal tree degradation in agricultural and forestry applications.

## Introduction

Stem borers are among the most destructive pests affecting a wide range of plant species, particularly in forestry and agricultural systems. The larvae penetrate plant tissues and feed internally, forming cavities within the stem that disrupt the structural integrity and physiological functions of the plant. In many cases, this internal damage remains undetected until visible symptoms appear, at which point the plant may already be severely weakened or irreversibly damaged. Therefore, early detection of internal defects is essential for effective plant protection, yield preservation, and sustainable agricultural management.

Conventional diagnostic techniques for plant health assessment, such as visual inspection or destructive sampling, are either unreliable for early-stage detection or impractical for large-scale monitoring. As a result, non-destructive evaluation (NDE) methods have attracted increasing attention for internal defect detection in biological materials. Among these, microwave sensing techniques have shown strong potential due to their ability to penetrate dielectric media and detect internal inhomogeneities based on variations in electromagnetic properties^1–3^.

The internal cavities generated by stem borer infestation create significant dielectric discontinuities within the plant tissues. These discontinuities perturb the propagation and scattering characteristics of electromagnetic waves, making them detectable using microwave-based sensing systems^4,5^. In particular, near-field microwave techniques are well suited for such applications, as they enable localized interaction with the medium and enhanced sensitivity to subsurface features^6^.

However, most existing antenna designs are primarily optimized for free-space radiation and standard communication applications^7–9^, and therefore do not account for operation in close proximity to lossy, heterogeneous media such as wood. When placed near biological tissues, conventional antennas experience strong impedance mismatch, current imbalance, and degraded radiation characteristics due to dielectric loading effects^7,8^. Furthermore, while advanced antenna concepts such as reconfigurable and adaptive structures have been extensively investigated for communication systems^10^, their application to medium-aware sensing in biological environments remains limited.

To address these challenges, this work proposes a medium-adaptive wideband near-field antenna specifically designed for operation in close proximity to plant stems. The antenna incorporates a compact quasi-planar dipole structure integrated with a dual-ring balun to ensure balanced current excitation and stable impedance matching under strong near-field loading conditions. Unlike conventional approaches, the antenna design explicitly accounts for the electromagnetic properties of the plant medium during the optimization process.

In this work, the term medium-adaptive refers to a passive antenna design strategy in which the antenna geometry and feeding structure are optimized to maintain stable electromagnetic performance when operating in close proximity to a strongly loading biological medium. Unlike actively reconfigurable antennas, the proposed design does not employ tunable components or dynamic impedance-control mechanisms. Instead, adaptation is achieved through a geometry specifically tailored to the dielectric characteristics of plant stems, enabling robust impedance matching, balanced current excitation, and high cavity-detection sensitivity despite variations in the surrounding medium properties.

In addition to antenna design, a conceptual two-element sensing configuration is introduced to evaluate the feasibility of detecting internal cavities using mutual coupling measurements. A sensitivity-driven framework is developed to quantify the response of the system to variations in cavity size and spatial location. This approach enables systematic identification of the optimal operating conditions for reliable detection.

The main contributions of this work can be summarized as follows: (i) Development of a medium-adaptive wideband near-field antenna for operation in lossy plant tissues. (ii) Introduction of a dual-ring balun structure to ensure balanced excitation under dielectric loading. (iii) Proposal of a two-element antenna system as a feasibility study for cavity detection. (iv) Formulation of a sensitivity analysis framework for evaluating detection performance. (v) Experimental validation of the antenna performance and sensing capability.

The remainder of this paper is organized as follows. Section  2 generally presents the problem of plant stem borers. Section  3 describes the proposed antenna design. Section  4 presents the electromagnetic modeling of the plant stem, including its dielectric properties and geometrical representation used in the design process. Section  5 presents the results of antenna parametric study as the methodology of design optimization under realistic loading conditions. Section 6 describes the medium-adaptive behavior of the proposed antenna. In Sect. 7 , the theoretical framework for cavity detection is introduced, including the formulation of the sensitivity metrics. Section  8 provides comprehensive simulation results, covering antenna performance, sensitivity analysis, and detection capability using the proposed two-element configuration. Section  9 describes the experimental work. Section  10 provides comparative performance evaluation with published related work. Section  11 is dedicated for discussing the achievements of the present work demonstrating its major limitations and presents the possible future work. Finally, Sect.  12 concludes the paper and outlines directions for future work.

### The problem of stem borers

Stem borers are among the most destructive pests affecting many plant species. The larvae penetrate plant tissues and feed internally, causing structural damage that can severely weaken or eventually kill the plant. In particular, the larvae bore into the stem and carve internal cavities while feeding and moving through the pith region.

**Fig. 1:**
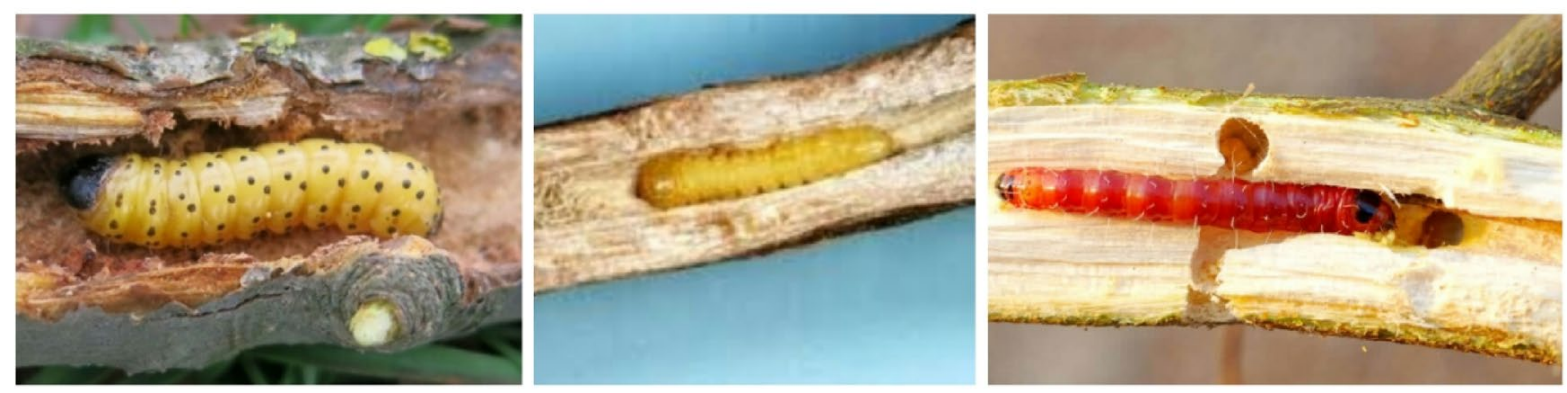
Three examples of stem infestation caused by stem borer larvae. The larvae penetrate the stem and create internal quasi-cylindrical cavities while feeding and moving through the plant tissues^11–13^.

As the larvae grow, they create elongated quasi-cylindrical cavities inside the stem, as illustrated in Fig. 1. These cavities disrupt the internal structure of the stem and hinder the transport of water and nutrients, which may ultimately lead to plant death.

A fully grown larva typically reaches a length of approximately 20 mm and is most commonly characterized by a reddish-brown head and a white body that may exhibit black spots.

**Fig. 2:**
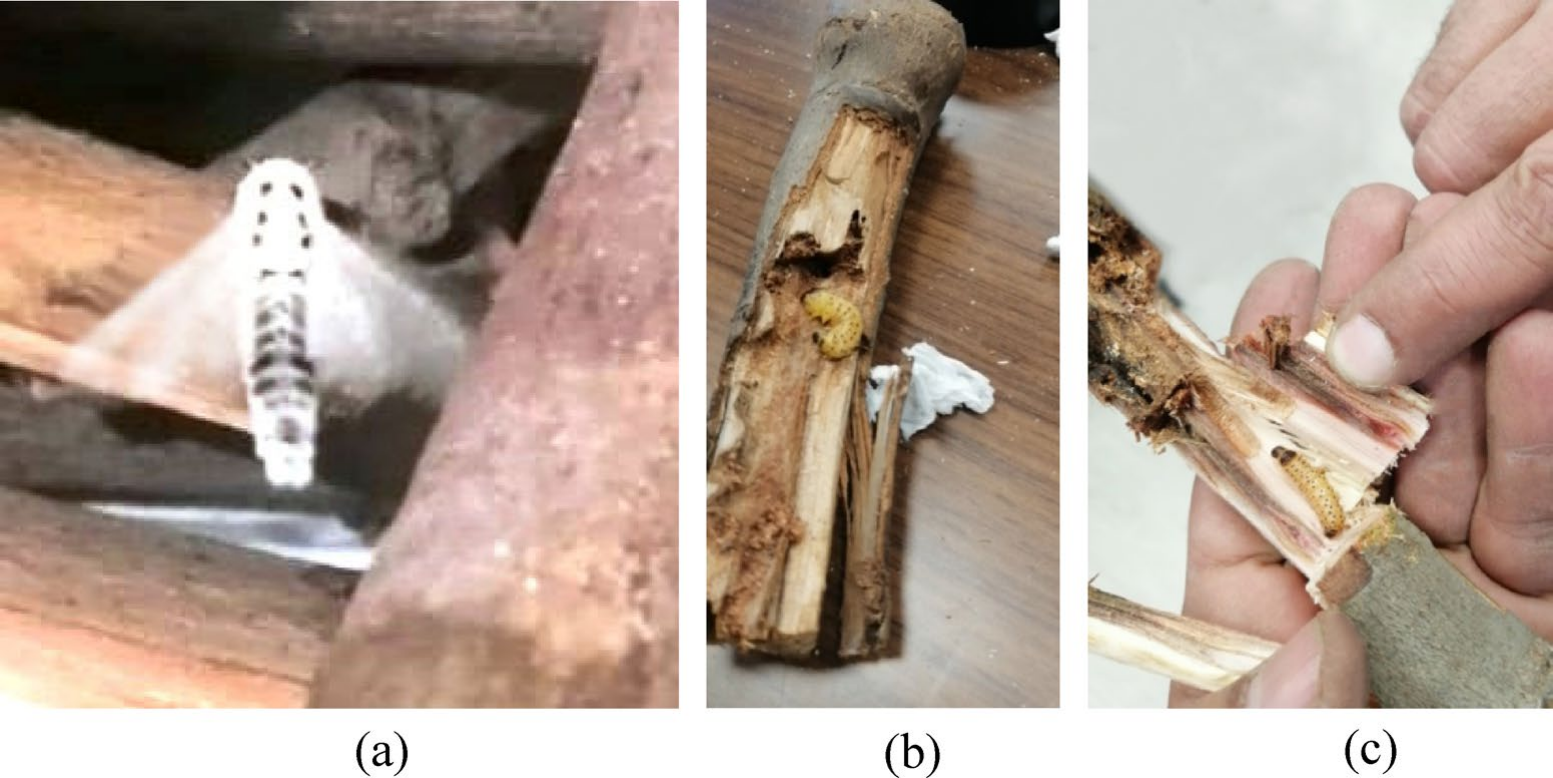
Samples of infested willow stems collected by the authors from the El-Fayoum region in Egypt. (**a**) Adult moth of the stem borer (**b**) Infested willow stem showing external damage caused by larval activity (**c**) Dissected stem revealing internal cavities formed by larval feeding and movement within the stem pith.

Figure 2 presents samples of infested stems of willow trees collected by the authors from the El-Fayoum region in Egypt. These samples were used for both simulation model development and experimental validation of the proposed detection system. The adult insect is a moth, shown in Fig. 2(a), with a wingspan ranging from approximately 8 to 15 mm and predominantly white wings. The eggs are deposited in clusters on plant leaves and are typically yellow in color.

**Fig. 3:**
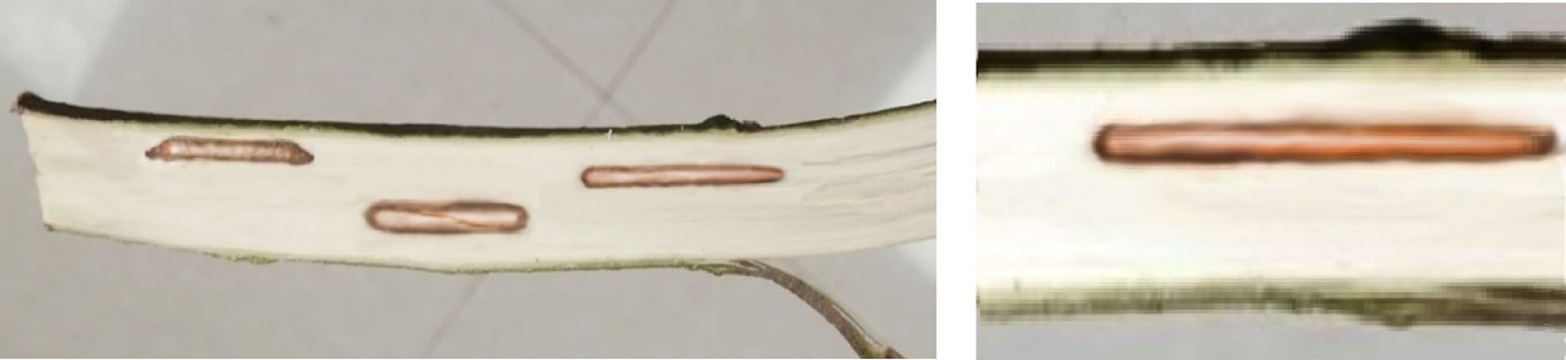
Dissected plant stems showing internal infestation. (a) Multiple cylindrical-shaped cavities of different sizes and locations inside the stem. (b) Enlarged view of one cavity confirming the quasi-cylindrical geometry produced by larval tunneling.

The collected stems exhibit clear evidence of internal damage caused by larval activity. In particular, large cavities and internal tunnels are observed within the stem tissues. To better examine the internal structure of the infestation, several stems were dissected longitudinally, as shown in Fig. 3. The dissection reveals multiple cavities with different sizes and spatial locations inside the stem. A magnified view of one of these cavities confirms their approximately quasi-cylindrical geometry, which is consistent with the feeding and movement patterns of the larvae.

The cavities produced by stem borer larvae introduce pronounced dielectric discontinuities within the plant tissues. These inhomogeneities perturb the propagation and scattering of microwave fields inside the stem, generating measurable electromagnetic signatures. Consequently, the detection of such defects represents a suitable application for microwave-based non-destructive sensing techniques. This requirement motivates the development of a carefully designed wideband near-field antenna capable of efficiently coupling electromagnetic energy into the wood tissues while maintaining high sensitivity to internal scattering mechanisms.2. Antenna Design.

## Antenna design

### Objectives and operational conditions

The proposed antenna is intended for near-field microwave sensing of plant stems. Since the antenna is positioned in very close proximity to the curved surface of the stem, the electromagnetic properties of the wood significantly influence antenna performance. Therefore, the lossy, anisotropic, and dispersive characteristics of the stem tissues must be incorporated into the design and simulation process.

The main design objectives are summarized as follows:

(i) The antenna impedance must be matched to a 50 Ω source over the frequency band 2–3 GHz.
(ii) The antenna geometry must be sufficiently compact to enable the deployment of multiple elements around the stem to form a radar-based inspection system.
(iii) The antenna should preferentially direct electromagnetic radiation into the tissues rather than radiating into free space.
(iv) The antenna reflection coefficient (self S-parameter) should exhibit sensitivity to near-surface inhomogeneities such as cavities and decayed regions.
(v) The mutual coupling (mutual S-parameters) between adjacent antennas should be sensitive to the size and lateral position of embedded inhomogeneities within the, regardless of their depth below the surface.

### Antenna placement and operating frequency

This section describes the placement configuration of the proposed antenna, the selected operating frequency band, and the environmental factors influencing performance during operation.

#### Antenna placement

The proposed antenna is designed as an element of a multi-antenna system arranged circumferentially around the stem. The overall configuration is conceptually similar to ground-penetrating radar (GPR) system, where electromagnetic waves are launched into the medium and the scattered fields are analyzed to detect internal anomalies.

Each antenna element is positioned very close to the stem surface to ensure strong electromagnetic coupling into the wood tissues. Direct contact with the stem surface is avoided to prevent impedance detuning caused by excessive loading.

During operation, the antenna transmits microwave energy into the stem tissues and receives the backscattered fields reflected from internal layers and inhomogeneous regions. Variations in the received signal indicate structural changes such as cavities or decay.

#### Operating frequency range

The selected operating frequency band is 2.0–3.0 GHz. This frequency range is chosen based on the following considerations:

(i) Microwaves in this band provide sufficient penetration depth into wood tissues while maintaining acceptable attenuation.
(ii) The corresponding wavelength inside wood enables detection resolution on the order of approximately $\:4\:\mathrm{m}\mathrm{m}$.
(iii) The band includes the unlicensed Industrial, Scientific, and Medical (ISM) band centered around 2.45 GHz.
(iv) Antenna dimensions at these frequencies remain compact, facilitating the construction of a multi-element array.

#### Environment and Role during Operation

Plant stems constitute a complex electromagnetic medium. Wood is an anisotropic material whose dielectric properties vary along the longitudinal, radial, and tangential directions of the tissues. This anisotropy influences wave velocity and propagation characteristics within the stem. Additionally, wood exhibits dispersive behavior, meaning that its dielectric properties vary with frequency. The medium is also lossy, with attenuation increasing significantly in the presence of moisture (wet wood compared to dry wood). All these properties strongly affect electromagnetic wave propagation and must be considered during antenna design and optimization.

In this work, the plant stem is modeled as a two-layer cylindrical structure consisting of the bark layer (outer region) and the pith (inner region). The detailed electromagnetic model is presented in Sect.  3.

### Antenna geometry and feeding

The proposed antenna is a balanced, two-arm blended-edge center-fed dipole. The antenna is fed using a coaxial cable through a matching balun to ensure proper transition between the unbalanced feed line and the balanced radiating structure. To minimize impedance detuning due to excessive loading, the antenna is positioned at a small clearance from the stem surface during operation.

#### Two-arm geometry

**Fig. 4:**
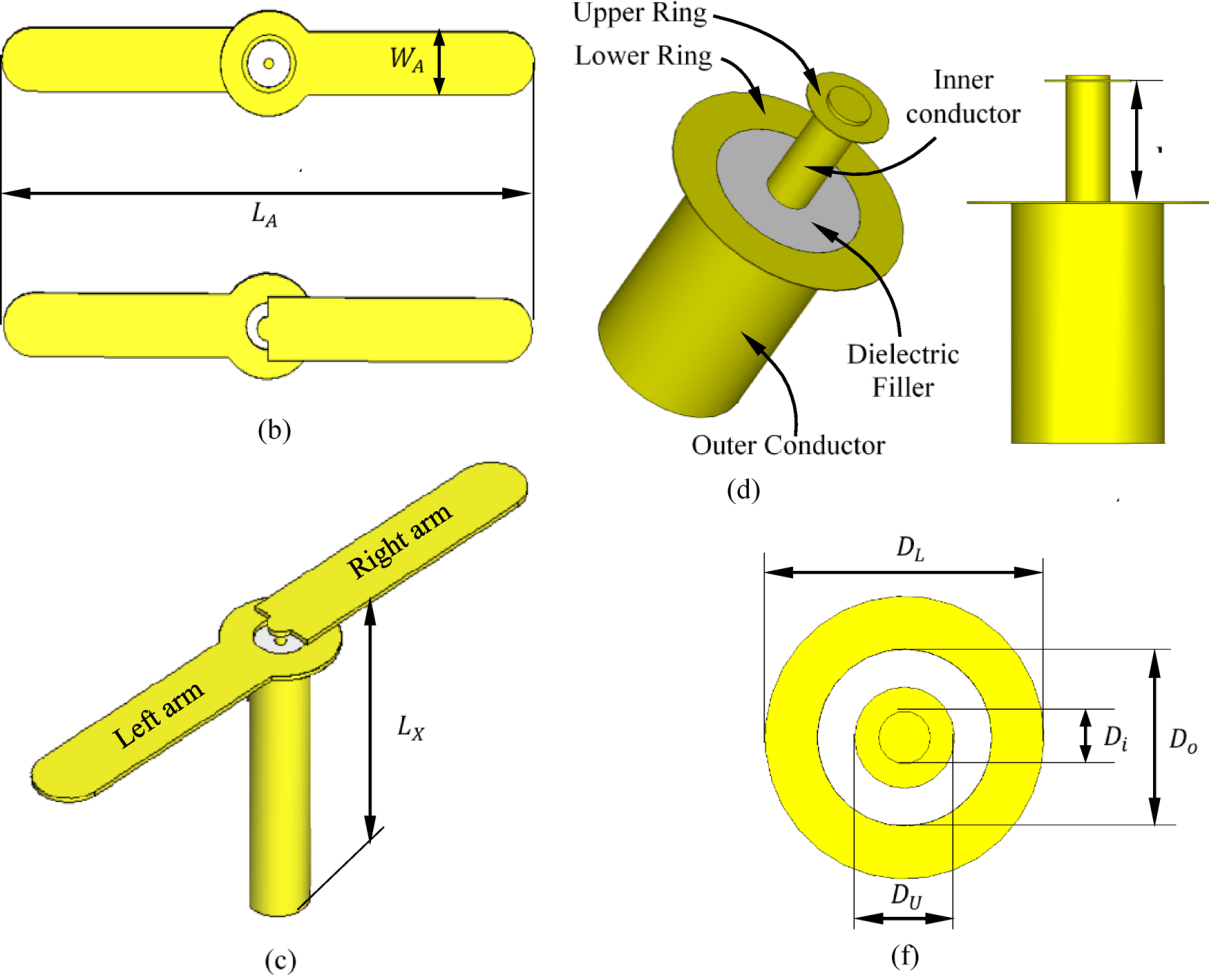
Structure and geometry of the antenna and feeding balun. (**a**) Bottom view of the antenna arms. (**b**) Top view of the antenna arms. (**c**) 3D view of the antenna. (**d**) 3D view of the balun. (**e**) Elevation view of the balun. (**f**) Top view of the balun showing the upper and lower rings.

The antenna consists of two wide arms with blended edges to achieve wideband impedance characteristics, as shown in Fig. 4. The arm length is denoted by $\:{L}_{A}$, and the arm width by $\:{W}_{A}$. The antenna is fed through a coaxial feed line of length $\:{L}_{X}$. The coaxial feed line consists of an outer conductor of diameter $\:{D}_{o}$, electrically connected to the left arm and an inner conductor of diameter $\:{D}_{i}$, connected to the right arm. The conductors are separated by a dielectric filler material with relative permittivity $\:{\epsilon\:}_{f}=2\:$and conductivity $\:{\sigma\:}_{f}=0$. This filler prevents short-circuiting between the conductors and helps control the characteristic impedance of the feeding structure. The optimal geometrical parameters are listed in Table 1.

#### Matching balun design

The antenna is inherently a balanced structure, whereas the coaxial feed line is unbalanced. Without proper transition, uneven current distribution would occur across the two arms due to the asymmetry between the inner and outer conductors of the coaxial line. This imbalance can lead to impedance mismatch, radiation pattern distortion, and reduced radiation efficiency.

To address this issue, a balun (balanced-to-unbalanced transformer) is introduced between the feed line and the antenna arms. The balun geometry is inspired by previously reported wideband balun designs [14]. It consists of two toroidal conductive rings, the upper ring, of diameter $\:{D}_{U}$, connected to the inner conductor, and the lower ring, of diameter $\:{D}_{L}$, connected to the outer conductor. The two rings are separated by a vertical distance $\:h$, as illustrated in Fig. 4.

This structure redistributes the electromagnetic fields at the feed transition region, equalizing the current distribution on both arms and improving wideband impedance matching.

The novelty of the proposed balun lies in its dual-toroidal ring configuration specifically optimized for near-field sensing in anisotropic and dispersive media. Unlike conventional coaxial-to-dipole transitions that assume free-space conditions, the present design accounts for strong dielectric loading from the plant stem and actively redistributes the electromagnetic fields at the feed region. This results in improved current symmetry, reduced common-mode excitation, and stable wideband impedance matching (2–3 GHz) even when the antenna operates in close proximity to a lossy cylindrical medium.

**Table 1:** Geometrical dimensions of two arm blended-edge center-fed dipole and matching balun shown in Fig. 4.

Parameter	$\:{\boldsymbol{L}}_{\boldsymbol{A}}$	$\:{\boldsymbol{W}}_{\boldsymbol{A}}$	$\:{\boldsymbol{D}}_{\boldsymbol{o}}$	$\:{\boldsymbol{D}}_{\boldsymbol{i}}$	$\:\boldsymbol{h}$	$\:{\boldsymbol{L}}_{\boldsymbol{X}}$	$\:{\boldsymbol{D}}_{\boldsymbol{L}}$	$\:{\boldsymbol{D}}_{\boldsymbol{U}}$
Value (mm)	$\:50$	$\:7$	$\:4.12$	$\:1.27$	$\:3$	$\:30$	$\:12$	$\:4$

It should be emphasized that the objective of the proposed dual-ring balun is not to replace conventional balun topologies, but rather to provide a compact balancing structure that can be readily integrated with the proposed near-field sensing antenna operating in close proximity to a heavily dielectric-loaded stem medium. In contrast to quarter-wavelength sleeve baluns or ferrite-based choke baluns, the proposed design consists only of two conductive rings directly connected to the coaxial feed, resulting in a compact geometry suitable for multi-element deployment around small plant stems. The effectiveness of the balun is demonstrated through the nearly symmetric current distributions observed on the two antenna arms over the entire operating band, as discussed later in Sect.  8.1.

### Plant stem model

To accurately evaluate the antenna performance under realistic operating conditions, a plant stem model was developed using CST Microwave Studio. The objective was to construct an electromagnetic representation that closely approximates the structural and dielectric characteristics of an actual plant stem, including material losses, anisotropy, and directional dependence of electrical properties.

### Geometrical modeling of plant stem

**Fig. 5:**
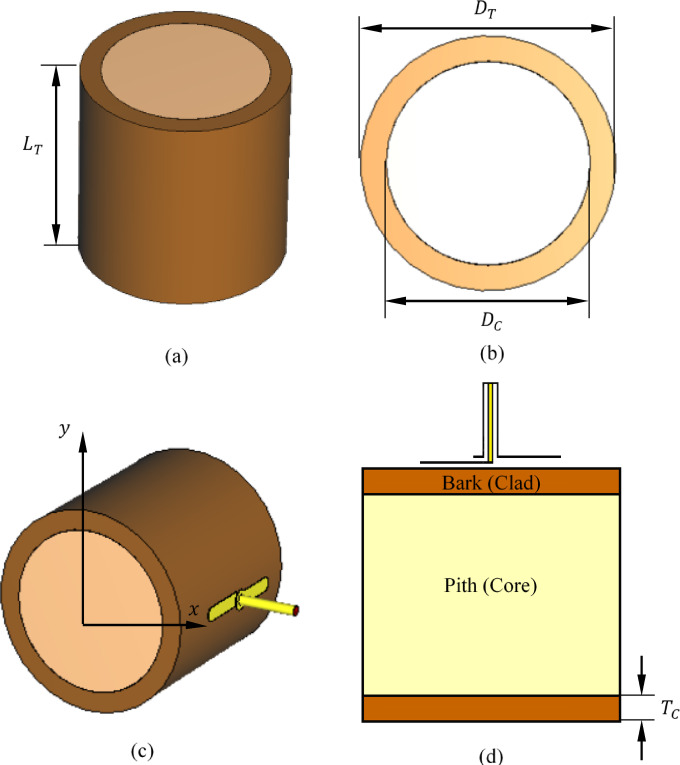
Model of plant stem. (**a**) 3D view. (**b**) Front view. (**c**) 3D view showing the proposed antenna on the stem model. (**d**) $\:yz$ cross-section showing the antenna on the stem model.

The plant stem is modeled as a two-layer cylindrical structure consisting of an outer bark layer (hard clad) and an inner pith region (soft core) as shown in Fig. 5. For electromagnetic simulation purposes, these regions are represented as two homogeneous dielectric layers with different electrical properties. The stem model has cylindrical shape of length $\:{L}_{T}$, the bark is defined as a tube of outer diameter $\:{D}_{T}$. The pith is modeled as a homogeneous cylinder of diameter $\:{D}_{C}$. The geometrical parameters of the stem model are listed in Table 2. This layered configuration allows the investigation of wave propagation and scattering behavior in a heterogeneous cylindrical medium while maintaining computational feasibility.

It should be noted that the adopted two-layer cylindrical stem model represents a first-order approximation intended to capture the dominant electromagnetic characteristics governing wave propagation, antenna loading, mutual coupling, and cavity scattering, rather than the full anatomical complexity of real plant stems. By incorporating bark and internal tissue layers, dielectric losses, anisotropy, and frequency-dependent behavior, the model adequately represents the principal mechanisms underlying microwave-based cavity detection.

Although real stems may exhibit irregular geometries, growth rings, moisture gradients, vascular structures, cracks, knots, and other forms of tissue heterogeneity, the proposed sensing approach relies primarily on the bulk dielectric contrast between healthy wood and cavity regions, such that the dominant scattering mechanisms are governed by the effective electromagnetic properties of the stem rather than by small-scale anatomical features.

Furthermore, sensitivity analyses conducted over a wide range of stem dielectric parameters demonstrated that the proposed antenna maintains stable impedance matching and cavity-detection sensitivity despite substantial variations in electrical properties, indicating that the main conclusions are robust to moderate deviations from the simplified stem model. The validity of this modeling approach is further supported by the good agreement observed between simulation and experimental measurements on real plant stems. Nevertheless, investigation of more realistic biological structures and large-scale field validation will be considered in future work.

**Table 2:** Dimensions of the plant stem model.

Parameter	$\:{\boldsymbol{D}}_{\boldsymbol{T}}$	$\:{\boldsymbol{L}}_{\boldsymbol{T}}$	$\:{\boldsymbol{T}}_{\boldsymbol{C}}$	$\:{\boldsymbol{D}}_{\boldsymbol{C}}$
Value (mm)	$\:100$	$\:100$	$\:10$	$\:90$

### Electromagnetic modeling of plant stem

Plant stem tissues exhibit frequency-dependent (dispersive), lossy, and anisotropic electrical behavior. To represent these characteristics, the dielectric properties of both layers are defined using realistic permittivity and conductivity values, as summarized in Table 3. The clad layer is modeled as an isotropic lossy dielectric characterized by relative permittivity $\:{\epsilon\:}_{r}$ and conductivity $\:\sigma\:$. In contrast, the core layer is modeled as an anisotropic medium using a tensor-based material definition. The permittivity and conductivity are expressed as diagonal tensors:

1 $$\: \mathop \varepsilon \limits^{ = } _{r} = \left( {\begin{array}{*{20}c} {\varepsilon _{{rxx}} } & 0 & 0 \\ 0 & {\varepsilon _{{ryy}} } & 0 \\ 0 & 0 & {\varepsilon _{{rzz}} } \\ \end{array} } \right)\varepsilon _{0} ,{\text{ }}\mathop \sigma \limits^{ = } = \left( {\begin{array}{*{20}c} {\sigma _{{xx}} } & 0 & 0 \\ 0 & {\sigma _{{yy}} } & 0 \\ 0 & 0 & {\sigma _{{zz}} } \\ \end{array} } \right)$$

This formulation accounts for the directional dependence of the wood tissues along the longitudinal, radial, and tangential axes. Such anisotropy significantly affects wave velocity, attenuation, and field distribution inside the stem. By incorporating tensor-based anisotropic and lossy dispersive material properties, the model provides a more realistic electromagnetic response compared to conventional isotropic approximations. This improves the reliability of simulated scattering parameters and ensures accurate prediction of antenna performance under near-field loading conditions.

Considering dispersion with frequency, the stem tissues are modeled in the present study using a frequency-dependent single-pole Debye formulation with conductive loss,

2 $$\:{\epsilon}\:^{*} \left( {\omega \:} \right) = {\epsilon}\:_{{\infty \:}} + \frac{{{\epsilon}\:_{s} - {\epsilon}\:_{{\infty \:}} }}{{1 + j\omega \:\tau \:}} - j\frac{{\sigma \:_{s} }}{{\omega \:{\epsilon}\:_{0} }}$$

The definitions and values of the Debye model parameters given by (2) are listed in Table 3.

**Table 3:** Parameters of the Debye model for the dispersive electric properties of the stem model layers.

Tissue Layers	Axis	Static Permittivity ($\:{\boldsymbol{\epsilon\:}}_{\boldsymbol{s}}$)	High-Frequency Permittivity ($\:{\boldsymbol{\epsilon\:}}_{\mathbf{\infty\:}}$)	Relaxation Time ($\:\boldsymbol{\tau\:}$)	Static Conductivity ($\:\boldsymbol{\sigma\:}\boldsymbol{s}$)
Clad	Isotropic	4	1.5	$\:12.0\:\mathrm{p}\mathrm{s}$	$\:0.085\:\mathrm{S}/\mathrm{m}$
Core	x-axis	12.4	4	$\:14.0\:\mathrm{p}\mathrm{s}$	$\:0.35\:\mathrm{S}/\mathrm{m}$
Core	y-axis	12.4	3.5	$\:14.0\:\mathrm{p}\mathrm{s}$	$\:0.20\:\mathrm{S}/\mathrm{m}$
Core	z-axis	24.2	3	$\:14.0\:\mathrm{p}\mathrm{s}$	$\:0.15\:\mathrm{S}/\mathrm{m}$

Wood moisture content is known to influence both dielectric permittivity and conductivity, thereby affecting microwave propagation and attenuation within plant tissues. Although the present study does not explicitly investigate seasonal moisture variations, robustness analyses performed under substantial dielectric-property variations demonstrated stable antenna operation and preserved cavity-detection sensitivity.

In practical deployments, measurements can be referenced to baseline responses acquired under the prevailing environmental conditions, allowing cavity detection to be performed using differential changes in the scattering parameters. Since the dielectric contrast between air-filled cavities and healthy wood is significantly larger than the variations typically introduced by moisture fluctuations, reliable cavity detection is expected to remain feasible under normal environmental conditions.

Definitely, the parameter values listed in Table 3 will vary with stem type and moisture content. However, the values listed in Table 3 are estimated at intermediate moisture for the most common types of the plants that are commonly subjected to stem borers.

**Fig. 6:**
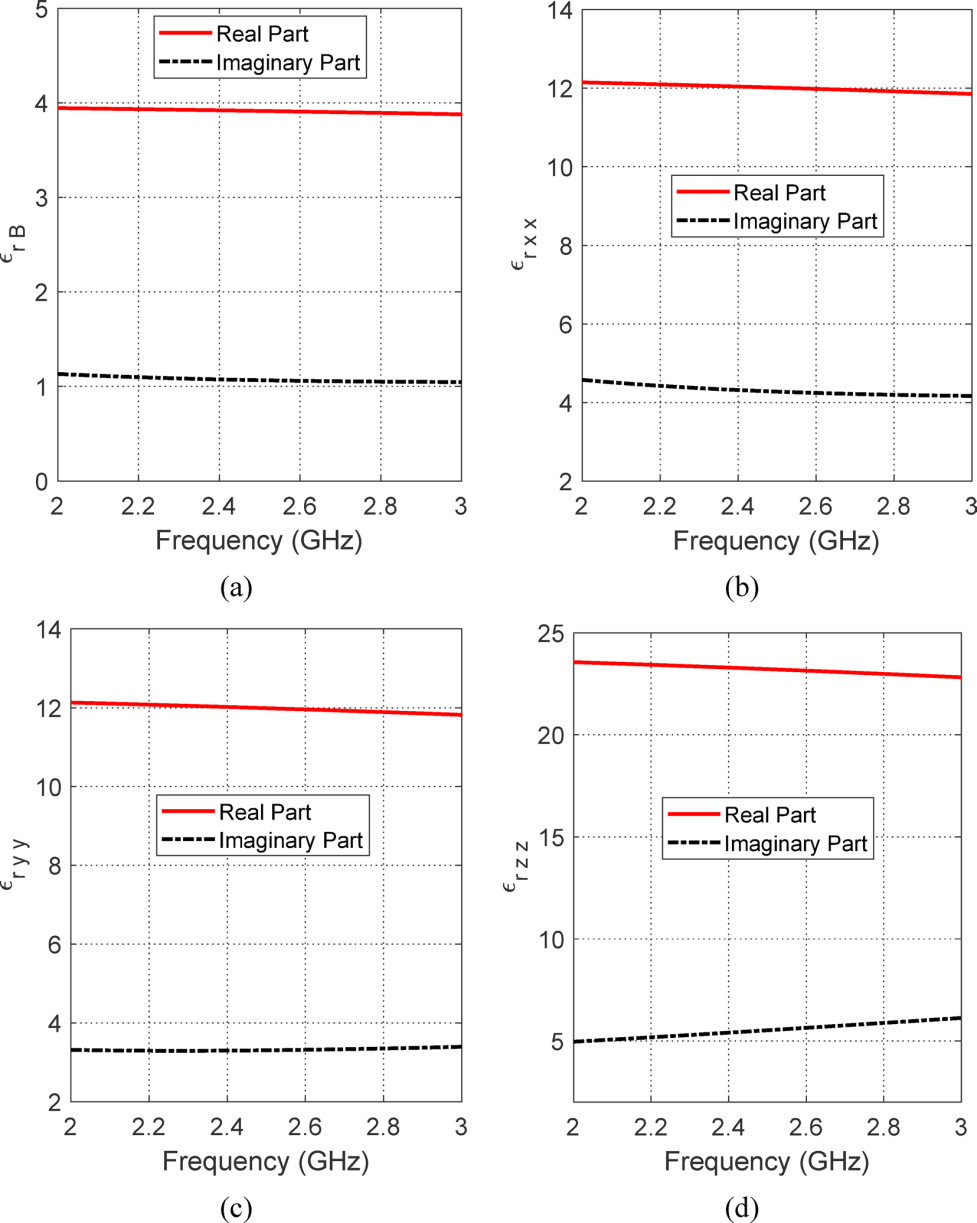
Simulation results for variation of the complex dielectric constant of the clad and core materials of the proposed plant stem model with the frequency. The Debye model (2) is used with the parameters listed in Table 3.

It is worth noting that the employed Debye parameters produce only a relatively weak dielectric variation across the investigated band as shown in Fig. 6. For example, the real part of the core permittivity along the $\:x$-direction varies by only a few percent between 2 and 3 GHz.

Similar gradual variations are observed for the other tensor components and for the bark layer. Therefore, the stem material exhibits only mild dispersion over the operating band. Table 4 shows the physical properties of the plant stem two-layer model and the dielectric properties at the middle frequency of operation (2.5 GHz).

**Table 4:** Physical properties of the stem model at 2.5 GHz.

Tissue layers	Density, $\:\boldsymbol{\rho\:}$ ($\:\mathbf{k}\mathbf{g}/{\mathbf{m}}^{3})$	Relative Permittivity ($\:{\boldsymbol{\epsilon\:}}_{\boldsymbol{r}}$)	Conductivity, $\:\boldsymbol{\sigma\:}\:(\mathbf{S}/\mathbf{m})$
Clad	$\:450$	$\:{\epsilon\:}_{r}=4$	$\:\sigma\:=0.15$
Core	$\:600$	$\:{\epsilon\:}_{xx}=12.0$	$\:{\sigma\:}_{xx}=0.60$
		$\:{\epsilon\:}_{yy}=12.0$	$\:{\sigma\:}_{yy}=0.46$
		$\:{\epsilon\:}_{zz}=23.3$	$\:{\sigma\:}_{zz}=0.77$

As discussed above (Fig. 6), both permittivity and conductivity exhibit only weak frequency dependence over the investigated band. Therefore, the optimum sensitivity frequency around 2.76 GHz is not a consequence of strong material dispersion, but rather of the electromagnetic interaction between the antenna near fields and cavity-induced perturbations within the stem.

### Role of the stem model in antenna optimization

The two-layer anisotropic cylindrical model is used during the antenna optimization process to determine the optimal geometrical parameters under realistic dielectric loading. Because the antenna operates in very close proximity to the surface, the electromagnetic interaction between the antenna and the medium strongly influences impedance matching, radiation distribution, and scattering behavior.

By performing full-wave simulations with the antenna mounted on the stem model as shown in Figs. 5(c) and 5(d), the proposed design is optimized to:

(v) Maintain wideband impedance matching (2–3 GHz),
(vi) Concentrate electromagnetic energy into the tissues,
(vii) Enhance sensitivity of both self and mutual S-parameters to internal inhomogeneities.

This medium-aware modeling approach distinguishes the present work from designs optimized solely in free-space conditions.

### Parametric study for optimization antenna design

Determining the optimal dimensions of the antenna and the balun (shown in Fig. 4) is an essential requirement for achieving high antenna performance when placed on the stem model (Sect.  4). Therefore, the effects of varying the dimensions of the antenna arms and the balun on the self-scattering parameter $\:\left|{S}_{11}\right|$ and impedance matching over the desired frequency band $\:2.0-3.0\:\mathrm{G}\mathrm{H}\mathrm{z}$ are studied using CST simulation.

### Parametric study for optimum dimensions of antenna arms

**Fig. 7:**
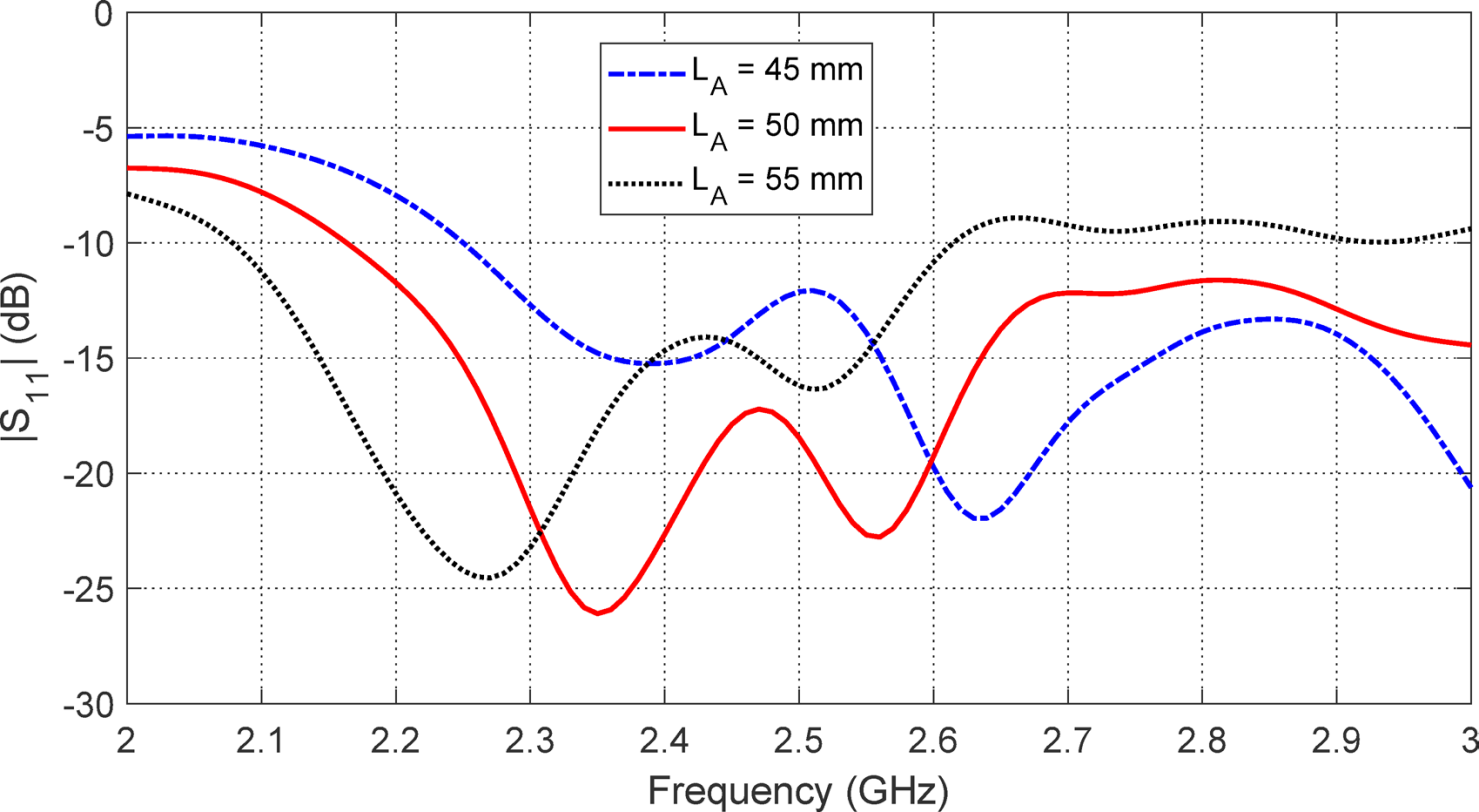
Simulation results for effect of center-fed dipole length on the frequency response of $\:\left|{S}_{11}\right|$, the optimum length of antenna at $\:{L}_{A}=50\:\mathrm{m}\mathrm{m}$.

**Fig. 8:**
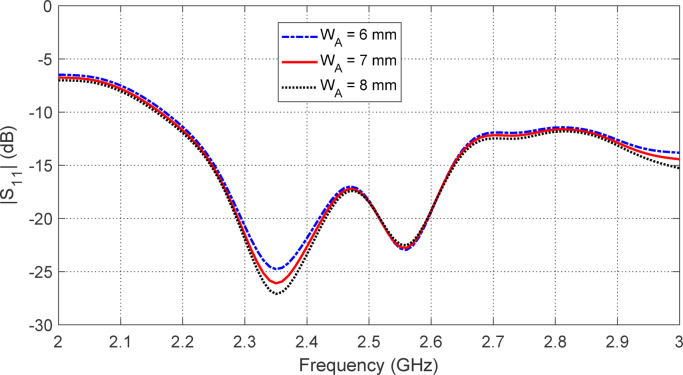
Simulation results for effect of the center-fed dipole width on the frequency response of $\:\left|{S}_{11}\right|$, the optimum width of antenna at $\:{W}_{A}=7\:\mathrm{m}\mathrm{m}$.

The optimum dimensions of the antenna were selected based on their effect on matching impedance over the operating frequency band. The dimensional parameters of the antenna arms that may affect the impedance matching over the frequency range are the antenna length $\:{L}_{A}$ and width $\:{W}_{A}$. Changing these parameters affects the frequency dependence of $\:\left|{S}_{11}\right|$ as shown in Figs. 7 and 8, respectively. The widest frequency band of impedance matching is obtained when $\:{L}_{A}=50\:\mathrm{m}\mathrm{m}$ as shown in Fig. 7. The arm width does not cause a significant variation like the antenna length as shown in Fig. 7. However, the selected arm width is $\:{W}_{A}=7\:\mathrm{m}\mathrm{m}$.

### Parametric study for optimum design of the matching balun

**Fig. 9:**
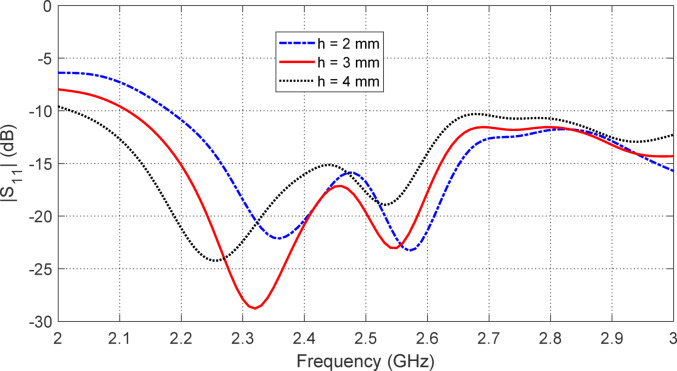
Simulation results for effect of the distance between two annular rings of the balun on the frequency response of $\:|{S}_{11}$|.

**Fig. 10:**
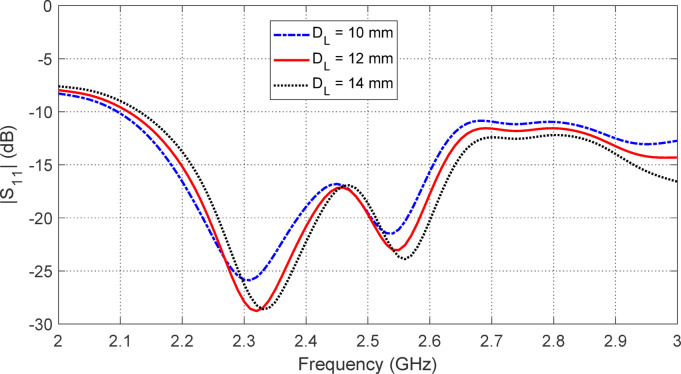
Simulation results for effect of the diameter of the lower annular ring of the balun on the frequency.

**Fig. 11:**
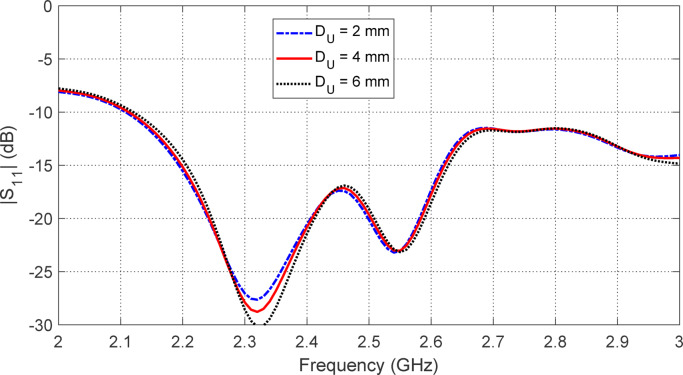
Simulation results for effect of the diameter of the upper annular ring of the balun on the frequency.

The antenna-designed balun can be optimized by adjusting its dimensions $\:h$, $\:{D}_{L}$and $\:{D}_{U}$ as shown in Table 1. The optimum dimensions achieve the best matching impedance between the antenna and the coaxial feed line over the desired frequency band. This is done using CST simulation to study the effect of changing these dimensions over the frequency band. From the frequency dependencies of $\:\left|{S}_{11}\right|$ shown in Figs. 9, 10 and 11, it is shown that the optimum performance regarding the impedance matching is achieved when the distance between two annular rings is $\:h=3$ mm, and the diameters of the lower and upper annular rings of the balun, respectively, are $\:{D}_{L}=12$ mm $\:{D}_{U}=4\:$ mm as.

response of $$\:|{S}_{11}$$|.

response of $$\:|{S}_{11}$$|.

### Medium adaptivity of the proposed antenna

To examine the medium adaptivity of the proposed antenna, its impedance matching performance is studied under wide variations in the electric properties of the plant stem. For this purpose, the anisotropic electric properties given by (1) can be expressed as follow.

3 $$\mathop {\varepsilon _{r} }\limits^{ = } = S_{\varepsilon } \mathop \varepsilon \limits^{{ = (0)}} _{r} ,{\text{}}\mathop \sigma \limits^{ = } = S_{\sigma } \mathop \sigma \limits^{{ = (0)}}$$

where, $\:{S}_{\epsilon\:}$ and $\:{S}_{\sigma\:}$ are scaling factors used to vary the permittivity and conductivity of the stem, respectively.

Accordingly, the anisotropic stem permittivity and conductivity tensors can be expressed as follows,

4 $$\:\epsilon\:={S}_{\epsilon\:}\left(\begin{array}{ccc}{\epsilon\:}_{rxx}^{\left(0\right)}&\:0&\:0\\\:0&\:{\epsilon\:}_{ryy}^{\left(0\right)}&\:0\\\:0&\:0&\:{\epsilon\:}_{rzz}^{\left(0\right)}\end{array}\right){\epsilon\:}_{0}$$

5 $$\:\sigma\:={S}_{\sigma\:}\left(\begin{array}{ccc}{{\upsigma\:}}_{xx}^{\left(0\right)}&\:0&\:0\\\:0&\:{{\upsigma\:}}_{yy}^{\left(0\right)}&\:0\\\:0&\:0&\:{{\upsigma\:}}_{zz}^{\left(0\right)}\end{array}\right)$$

For studying medium adaptivity of the proposed antenna, the scale factors $\:{S}_{\epsilon\:}$ and $\:{S}_{\sigma\:}$ are varied such that $\:{\stackrel{̿}{\epsilon\:}}_{r}$ and $\:\stackrel{̿}{\sigma\:}$ range from $\:75\%$ to $\:125\%$ of the nominal values $\:{\stackrel{̿}{\epsilon\:}}_{r}^{\left(0\right)}$ and $\:{\stackrel{̿}{\sigma\:}}^{\left(0\right)}$, respectively. This range of variation is sufficiently broad to assess the stability of antenna impedance matching over substantial changes in the stem electric properties. It should be noted that the nominal values of the electric properties, $\:{\stackrel{̿}{\epsilon\:}}_{r}^{\left(0\right)}$ and $\:{\stackrel{̿}{\sigma\:}}^{\left(0\right)}$, are selected because they are representative of the electrical properties of most common tree species.

The proposed antenna is characterized by its strong adaptability to the surrounding medium enabling reliable detection cavities inside various types of plant stems without degrading antenna performance. The following presentation of results demonstrates the medium adaptivity of the proposed antenna.

**Fig. 12:**
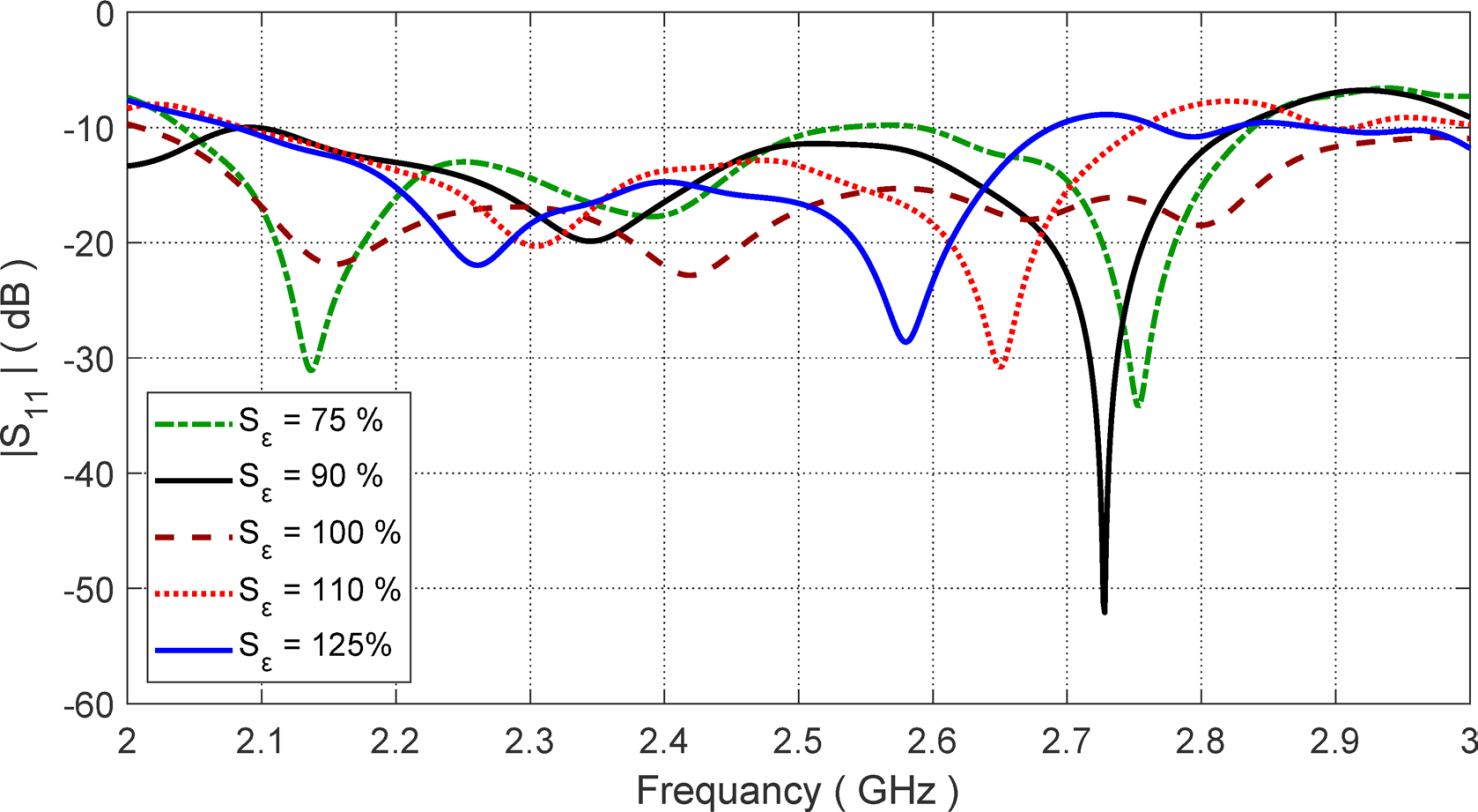
Simulation results for effect of frequency response of |$\:{S}_{11}$| for different value of permittivity scaling factor $\:{S}_{\epsilon\:}$ from 75% to 125% of the nominal values.

**Fig. 13:**
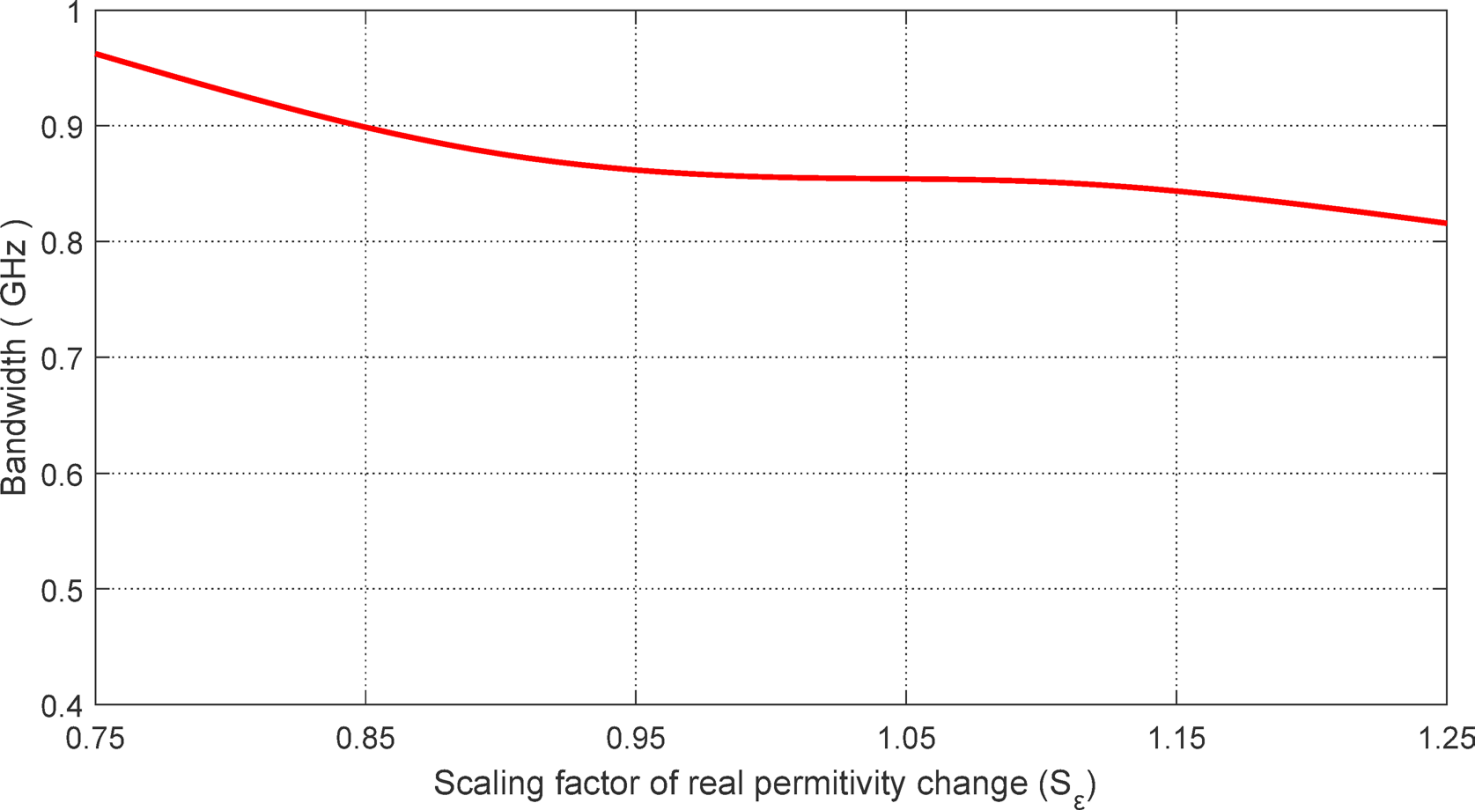
Simulation results for variation in bandwidth for different values of the permittivity scaling factor$\:\:{S}_{\epsilon\:}$, ranging from $\:75\%\:$to $\:125\%$ of the nominal values.

Figure 12 illustrates the effect of wide variations in the permittivity on the frequency response of $\:\left|{S}_{11}\right|$ over the operating band ($\:2-3\:\mathrm{G}\mathrm{H}\mathrm{z}$). This is achieved by varying the relative permittivity tensor $\:{\stackrel{̿}{\epsilon\:}}_{r}$ over the range $\:75\%\:$to $\:125\%$ of its nominal tensor $\:{\stackrel{̿}{\epsilon\:}}_{r}^{\left(0\right)}$. It can be observed that the proposed antenna preserves stable impedance matching bandwidth despite substantial changes in the permittivity of the stem, demonstrating its strong adaptability to different surrounding medium. Figure 13 shows the dependence of the bandwidth on the permittivity scaling factor$\:\:{S}_{\epsilon\:}$, ranging from $\:75\%$ to $\:125\%$. It is observed that the bandwidth is not significantly affected by changes in permittivity over such a wide range. Thus, the antenna is capable of maintaining wideband impedance matching in media with different permittivity, further confirming its medium adaptivity.

**Fig. 14:**
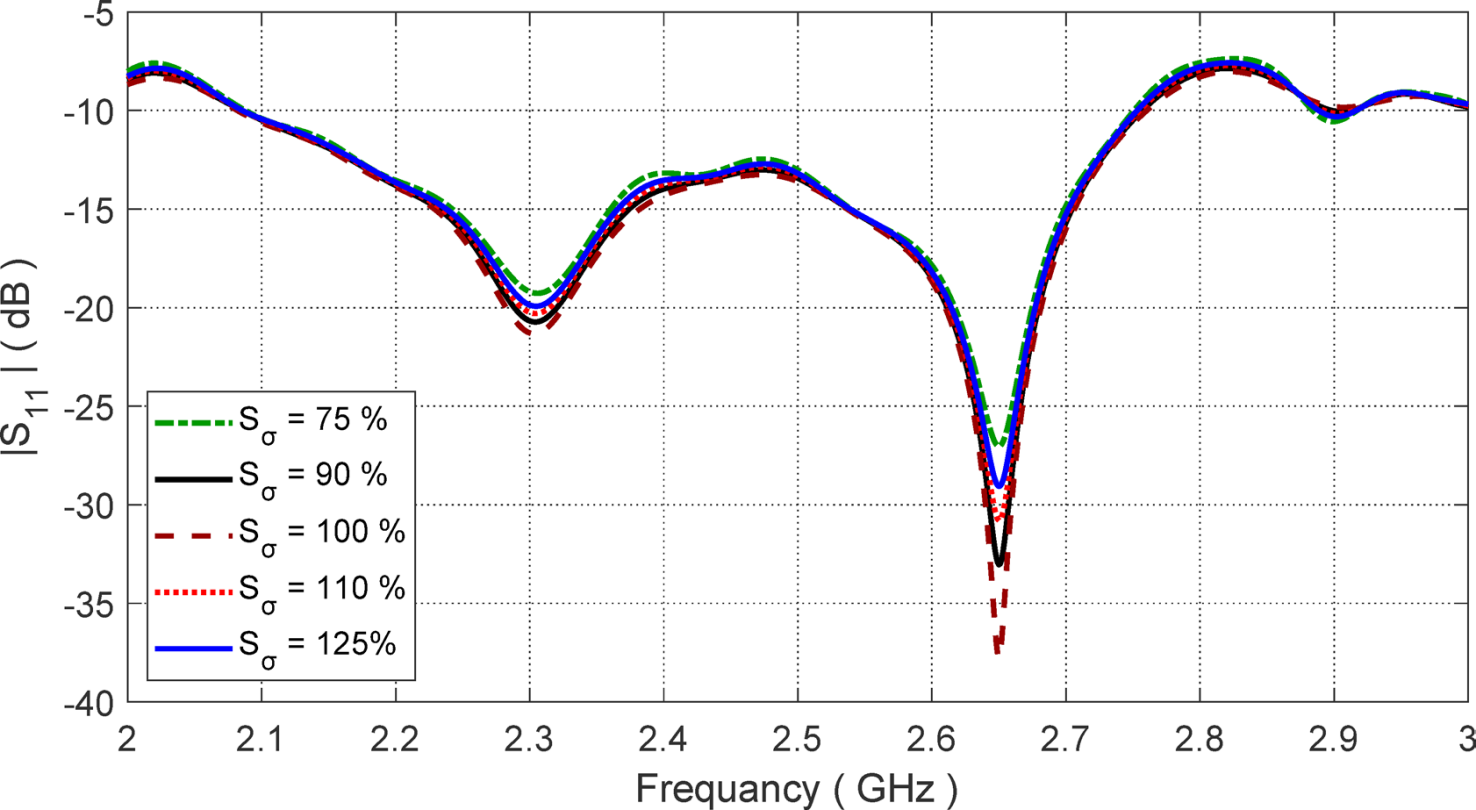
Simulation results for frequency response of |$\:{S}_{11}$| for different value of conductivity scaling factor $\:{S}_{\sigma\:}$ from 75% to 125% of the nominal values.

Figure 14 illustrate variation of frequency response of $\:\left|{S}_{11}\right|$ for different value of conductivity scaling factor $\:{S}_{\sigma\:}$ from $\:75\%$ to$\:\:125\%\:$of the nominal value. It can be observed that the antenna performance over frequency band is approximately constant. This indicates the proposed antenna’s capability to adapt to the surrounding medium and operate efficiently in environment with varying electric properties.

**Fig. 15:**
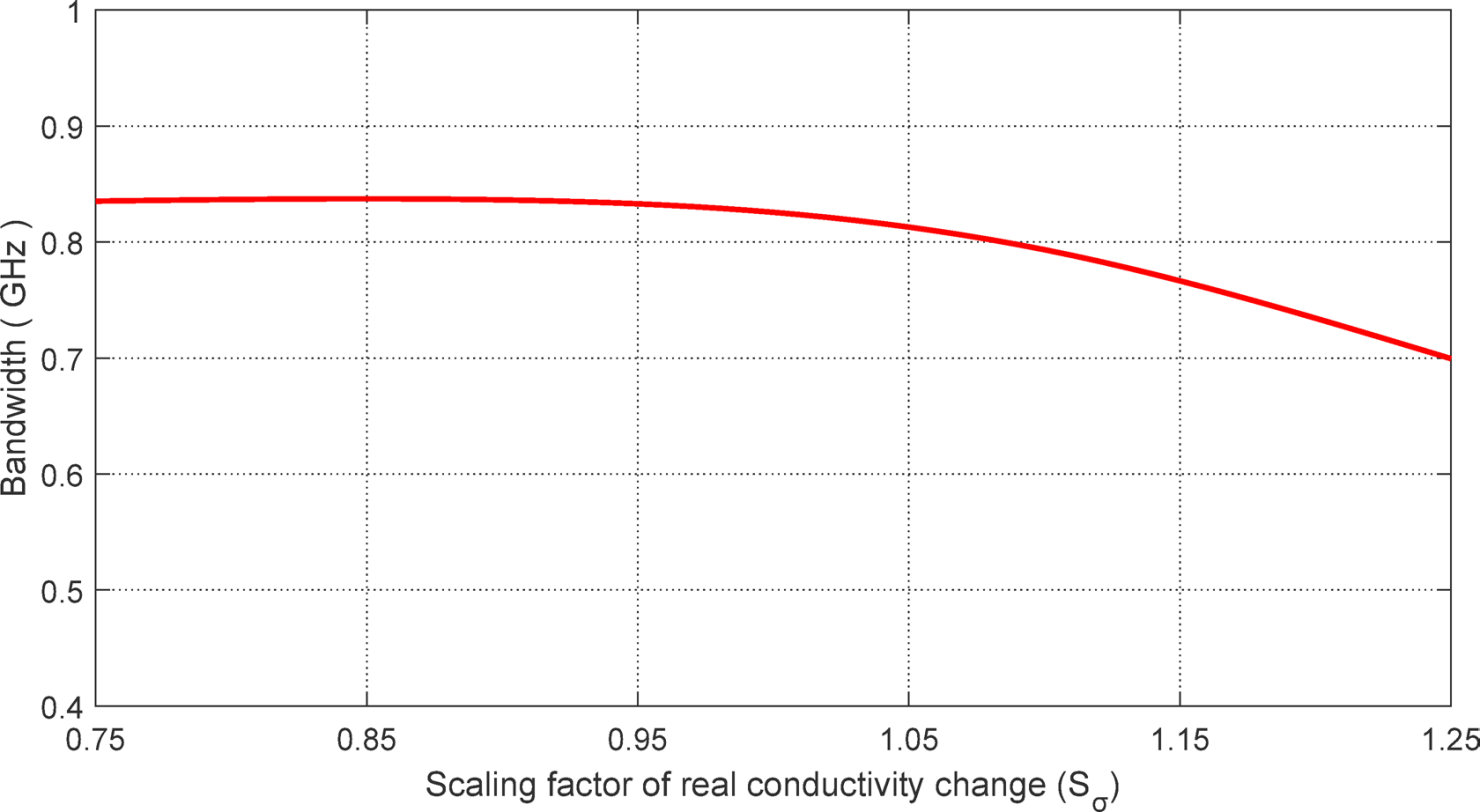
Simulation results for variation of bandwidth for different values of the conductivity scaling factor$\:\:{S}_{\sigma\:}$, ranging from $\:75\%$ to$\:\:125\%$ of nominal value.

Figure 15 presents the variation in bandwidth for different values of the conductivity scaling factor$\:\:{S}_{\sigma\:}$, ranging from $\:75\%$ t o $\:125\%$ of the nominal values. The result indicates that the bandwidth remains nearly constant over wide variation of conductivity variation, reflecting the antenna’s performance stability.

### A System of antenna pair: Minimum configuration for stem cavity detection

**Fig. 16:**
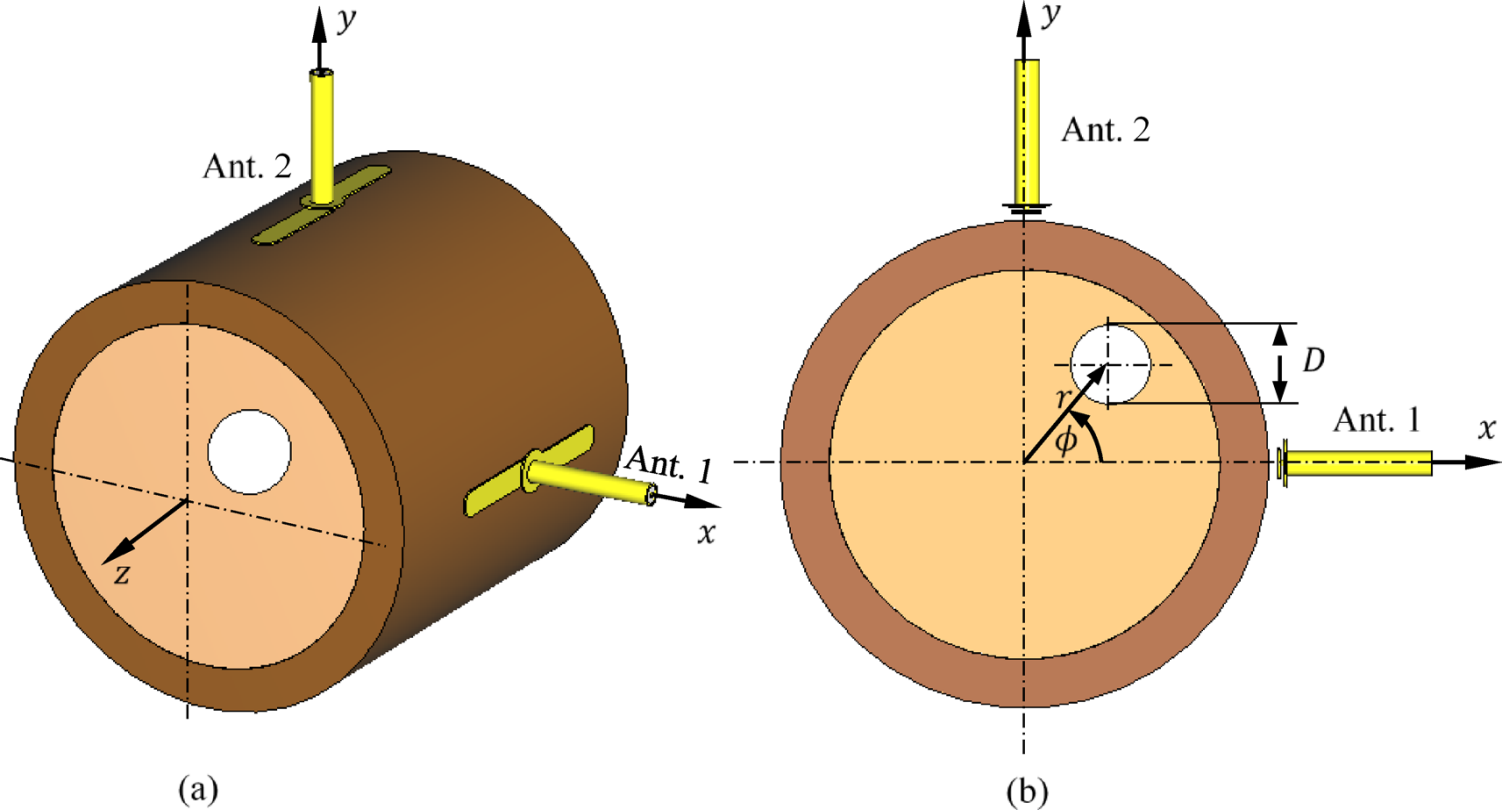
Conceptual stem cavity detection system formed by a pair of antennas mounted on the stem surface with a small clearance distance $\:d$. The angular positions of the two antennas are $\:\phi\:=0^\circ\:$ and $\:\phi\:=90^\circ\:$.

A simplified sensing configuration consisting of a pair of the proposed planar dipole antennas can be used to form a microwave radar unit for detecting cavities embedded within plant stems as a result of stem borer infestation provided that the cavity lies within the angular sector between the two antennas. The two antennas are mounted on the stem surface with a small clearance, as illustrated in Fig. 16. The angular positions of Ant. 1 and Ant. 2 are $\:\phi\:=0^\circ\:$ and $\:\phi\:=90^\circ\:$, respectively. In this configuration, one antenna primarily acts as a transmitting element while the other receives the scattered electromagnetic fields from the interior of the stem.

Variations in both the reflection coefficient (self-scattering parameter) and the transmission coefficient (mutual scattering parameter) of the antenna pair are exploited as indicators of the presence of internal inhomogeneities. In particular, changes in the diameter$\:D$. The geometry of this simplified detection configuration is shown in Fig. 10.

The antenna pair configuration represents the minimum sensing arrangement capable of detecting the existence of an embedded cavity and estimating its size of (diameter $\:D$), as illustrated in Fig. 16(b), provided that the cavity lies within the angular sector $\:0<\phi\:<90^\circ\:$ (i.e. between the two antennas).

The present work focuses on:

(i) Designing the proposed wideband antenna and evaluating its impedance matching performance.
(ii) Identifying the most suitable operating frequency band that maximizes the sensitivity of the mutual scattering parameter $\:{S}_{21}$ to variations in cavity diameter ($\:D$).
(iii) Performing experimental measurements to validate the simulation results related to antenna performance and sensing capability.

From a practical perspective, each antenna does not require direct contact with the stem surface. A clearance of $\:d=2\:\mathrm{m}\mathrm{m}\:$between the antenna and the stem surface can be maintained using a simple non-conductive spacer or mechanical support. Since the sensitivity varies gradually with clearance and acceptable performance is maintained over a range of small separations, moderate variations caused by bark roughness, stem curvature, or environmental motion are not expected to significantly affect the cavity-detection capability. This characteristic improves the suitability of the proposed sensing approach for future field deployment.

### Relationship between mutual coupling and cavity size

The mutual scattering parameter ($\:|S₂₁|$) measures the electromagnetic coupling between two antennas through the plant stem. In a healthy stem without defects, waves propagate through a homogeneous medium, producing a stable baseline response determined by the stem’s dielectric properties and geometry. However, the presence of internal cavities changes the dielectric distribution, disturbing the electromagnetic field and transmission paths. Consequently, variations in $\:|S₂₁|$ become dependent on the existence and size of the cavity $\:D$.

Under a first-order electromagnetic perturbation approximation, variations in the mutual coupling magnitude may be related to the permittivity perturbation introduced by the cavity through the relation,

6 $$\:\varDelta\:\left|{S}_{21}\right|\propto\:{\iiint\:}_{V}^{}\varDelta\:{\epsilon\:}_{r}\left(\mathbf{r}\right){\left|\mathbf{E}\left(\mathbf{r}\right)\right|}^{2}dV$$

where $\:V$ denotes the cavity volume and $\:\mathbf{E}\left(\mathbf{r}\right)$ is the unperturbed electric field distribution inside the stem.

Larger cavities introduce a stronger dielectric discontinuity, increasing scattering and attenuation within the propagation path, which results in a larger deviation of $\:\left|{S}_{21}\right|$ from its baseline value. Conversely, smaller cavities produce weaker perturbations, leading to more subtle changes in the mutual coupling magnitude.

These observations confirm that monitoring changes in the mutual scattering parameter $\:\left|{S}_{21}\right|$ enables reliable discrimination of cavity size within the plant stem, forming the basis for the sensitivity analysis presented in the subsequent section.

### Sensitivity analysis of mutual coupling to internal cavities

To quantitatively assess the capability of the proposed antenna system to detect internal defects, a sensitivity analysis is conducted based on variations in the magnitude of the mutual scattering parameter $\:\left|{S}_{21}\right|$. The analysis evaluates the response of $\:\left|{S}_{21}\right|$ to changes in cavity size ($\:D$).

The sensitivity metrics proposed in this work are formulated using the magnitude of the mutual scattering parameter, ($\:\left|{S}_{21}\right|$), because it provides a direct and experimentally robust measure of cavity-induced perturbations in the propagation channel. Although the phase component of ($\:\left|{S}_{21}\right|$) also contains information related to wave propagation and cavity location, the use of magnitude-only measurements enables straightforward interpretation of the antenna response and reduces sensitivity to measurement uncertainties.

The incorporation of complex-valued scattering parameters, including phase information, may further enhance cavity localization and parameter estimation accuracy and will be considered in future studies involving multi-element antenna systems and advanced inversion algorithms.

It should be noted that the expressions used in the following subsections (7.2.1 and 7.2.2) are introduced to provide physical interpretation of the dependence of the mutual coupling response on cavity-induced dielectric perturbations. They represent first-order qualitative approximations intended to explain the observed trends in sensitivity.

The quantitative results presented throughout this work are not derived from these expressions but are obtained from rigorous full-wave electromagnetic simulations using CST Microwave Studio. Therefore, the equations should be regarded as illustrative rather than predictive models.

#### Normalized differential sensitivity

The normalized differential sensitivity metric is defined as the ratio between the variation in the magnitude of the mutual scattering parameter $\:\left|{S}_{21}\right|$ resulting from a small change ($\:{\Delta\:}D$) in the cavity diameter around a reference value $\:\left({D}_{0}\right)$ and $\:{\Delta\:}D$. It is defined as,

7 $$\:{\widehat{S}}_{D}=\frac{\left|{S}_{21}({D}_{0}+{\Delta\:}D)\right|-\left|{S}_{21}\left({D}_{0}\right)\right|}{{\Delta\:}D}\:\mathrm{d}\mathrm{B}/\mathrm{m}\mathrm{m}$$

The normalized sensitivity provides a framework for evaluating and the relative impact of varying the cavity size on the electromagnetic response.

#### Electromagnetic interpretation using cavity scattering cross-section

The cavity inside the stem acts as a dielectric discontinuity that scatters part of the transmitted electromagnetic wave. This scattered field contributes to the received signal and changes the mutual coupling $\:|S₂₁|$ between the antennas. The variation in $\:|S₂₁|$ is proportional to the square root of the cavity scattering cross-section $\:{\sigma\:}_{S}\:$and inversely proportional to the distances between the cavity and the transmitting/receiving antennas:

8 $$\:\varDelta\:\left|{S}_{21}\right|\propto\:\frac{\sqrt{{\sigma\:}_{S}}}{{R}_{tc}{R}_{cr}}$$

This means that larger cavities or cavities located along the main propagation path produce stronger disturbances in the measured signal. For small cavities compared to the wavelength inside wood, the scattering cross-section approximately follows:

9 $$\:{\sigma\:}_{S}{\propto\:D}^{2}$$

Therefore, increasing cavity diameter $\:D$ leads to stronger variations in $\:|S₂₁|$.

### Simulation results and discussions

This section presents and discusses the simulation results used to assess the performance of the proposed antenna system. Unless otherwise stated, each antenna is placed at a clearance distance of d = 2 mm from the surface of the healthy stem model, whose geometry and electromagnetic properties are described in Sect.  4. The simulations are conducted over the frequency range of 2–3 GHz.

### Surface current distribution

**Fig. 17:**
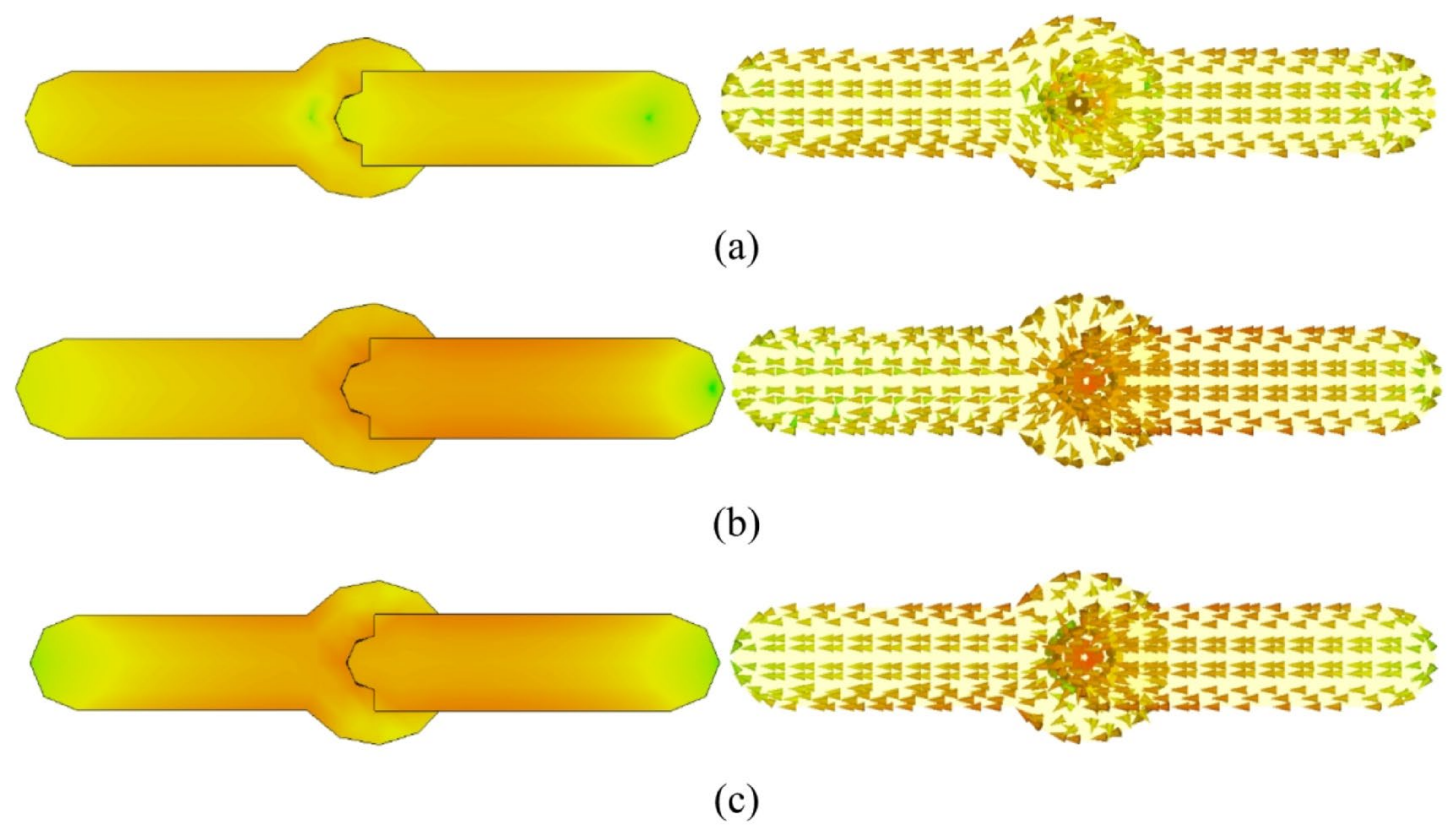
Simulation results for surface current distribution of the center-fed dipole antenna at different frequencies: (**a**) 2 GHz, (**b**) 2.5 GHz, and (**c**) 3 GHz. The antenna is positioned at a clearance distance of $\:d=2\:\mathrm{m}\mathrm{m}\:$from the surface of a healthy stem.

The surface current distribution provides important insight into the radiation behavior of the proposed antenna and the effectiveness of the integrated balun in maintaining balanced excitation of the dipole arms. Figure 17 illustrates the simulated surface current density on the antenna when positioned at a clearance distance of $\:d=2\:\mathrm{m}\mathrm{m}\:$from the surface of a healthy stem model. The current distribution is shown at three representative frequencies within the operational band of 2–3 GHz.

At all examined frequencies, the surface currents are predominantly concentrated along the dipole arms and gradually decay toward the arm extremities, which is characteristic of a center-fed dipole operating in the fundamental mode. More importantly, the current density distributions on the two arms remain nearly symmetric across the entire frequency band. This balanced current behavior indicates that the feeding structure effectively suppresses common-mode currents on the feed line.

The observed symmetry of the current distribution confirms the proper operation of the proposed balun structure, which ensures balanced excitation of the dipole arms even when the antenna operates in close proximity to the lossy stem medium. This balanced current distribution is essential for maintaining stable radiation characteristics and reliable sensing performance across the wide operating bandwidth.

### Distribution of the specific absorption rate (SAR) over the stem tissues

The interaction between the radiated electromagnetic fields and the biological tissues of the plant stem can be evaluated through the distribution of the specific absorption rate (SAR), which represents the rate at which electromagnetic energy is absorbed by the medium. Assessing the SAR distribution is important to ensure that the proposed sensing system operates at power levels that do not cause excessive electromagnetic energy deposition within the plant tissues.

**Fig. 18:**
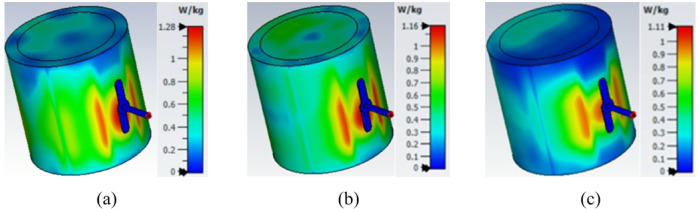
Simulation results for three-dimensional SAR distribution in the plant stem model at (**a**) 2 GHz, (**b**) 2.5 GHz, and (**c**) 3 GHz when the antenna input power is 8 dBm.

Figure 18 presents the simulated three-dimensional SAR distribution within a healthy stem model obtained using CST Microwave Studio. The results correspond to a single dipole antenna positioned at a clearance distance of $\:d=2\:\mathrm{m}\mathrm{m}\:$from the stem surface, with an input power of 8 dBm. The SAR distributions are shown at three representative frequencies within the operational band.

The maximum SAR values averaged over 10 g of tissue are found to be $\:1.28\:\mathrm{W}/\mathrm{k}\mathrm{g}$, $\:1.16\:\mathrm{W}/\mathrm{k}\mathrm{g}$ and $\:1.11\:\mathrm{W}/\mathrm{k}\mathrm{g}$ at frequencies of 2 GHz, 2.5 GHz, and 3 GHz, respectively. These relatively low values indicate that only a small portion of the transmitted electromagnetic energy is absorbed by the stem tissues. Consequently, the proposed sensing configuration operates at power levels that are unlikely to cause thermal or physiological stress to the plant.

The results therefore confirm that the proposed antenna-based sensing approach can be safely used for non-destructive monitoring of plant stems while maintaining adequate electromagnetic interaction with the internal tissues for reliable cavity detection.

### Radiation characteristics of the proposed antenna

The radiation characteristics in a free space are a secondary effect resulting from operation, as the purpose of the antenna is not to radiate in a free space. However, they are studied to determine the impact of radiation levels during the detection operation on the stem surroundings.

#### Radiation Patterns

**Fig. 19:**
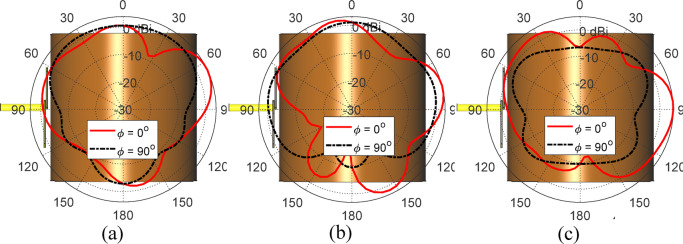
Simulation results for Gain pattern of the proposed antenna in the vertical planes $\:\phi\:=\:0$ and $\:\phi\:=\:90^\circ\:$ at (**a**) $\:2\:\mathrm{G}\mathrm{H}\mathrm{z}$. (**b**) $\:2.5\:\mathrm{G}\mathrm{H}\mathrm{z}$. (**c**) $\:3\:\mathrm{G}\mathrm{H}\mathrm{z}$.

**Fig. 20:**
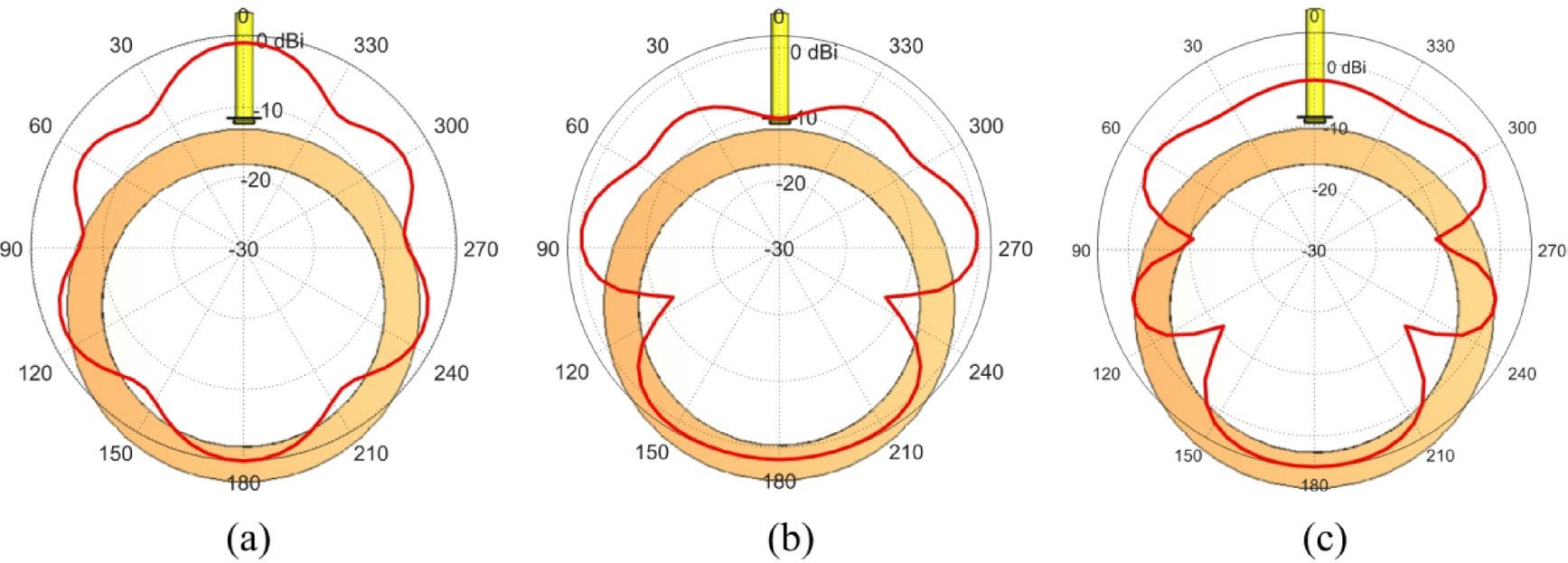
Simulation results for Gain pattern of the proposed antenna in the horizontal plane $\:\theta\:=90^\circ\:$ at (**a**) $\:2\:\mathrm{G}\mathrm{H}\mathrm{z}$. (**b**) $\:2.5\:\mathrm{G}\mathrm{H}\mathrm{z}$. (**c**) $\:3\:\mathrm{G}\mathrm{H}\mathrm{z}$.

The radiation pattern of proposed antenna while being placed on the stem model at a clearance $\:d=2\:\mathrm{m}\mathrm{m}$ in the elevation and azimuth planes are shown in Figs. 19 and 20, respectively. The purpose of investigating the far field patterns is to provide useful results that may be required for study the effects of the field radiated to the surrounding medium during the proposed detection process. This allows studying important issues such as electromagnetic radiation hazards, electromagnetic compatibility (EMC) and interference (EMI).

#### Gain and directivity

**Fig. 21:**
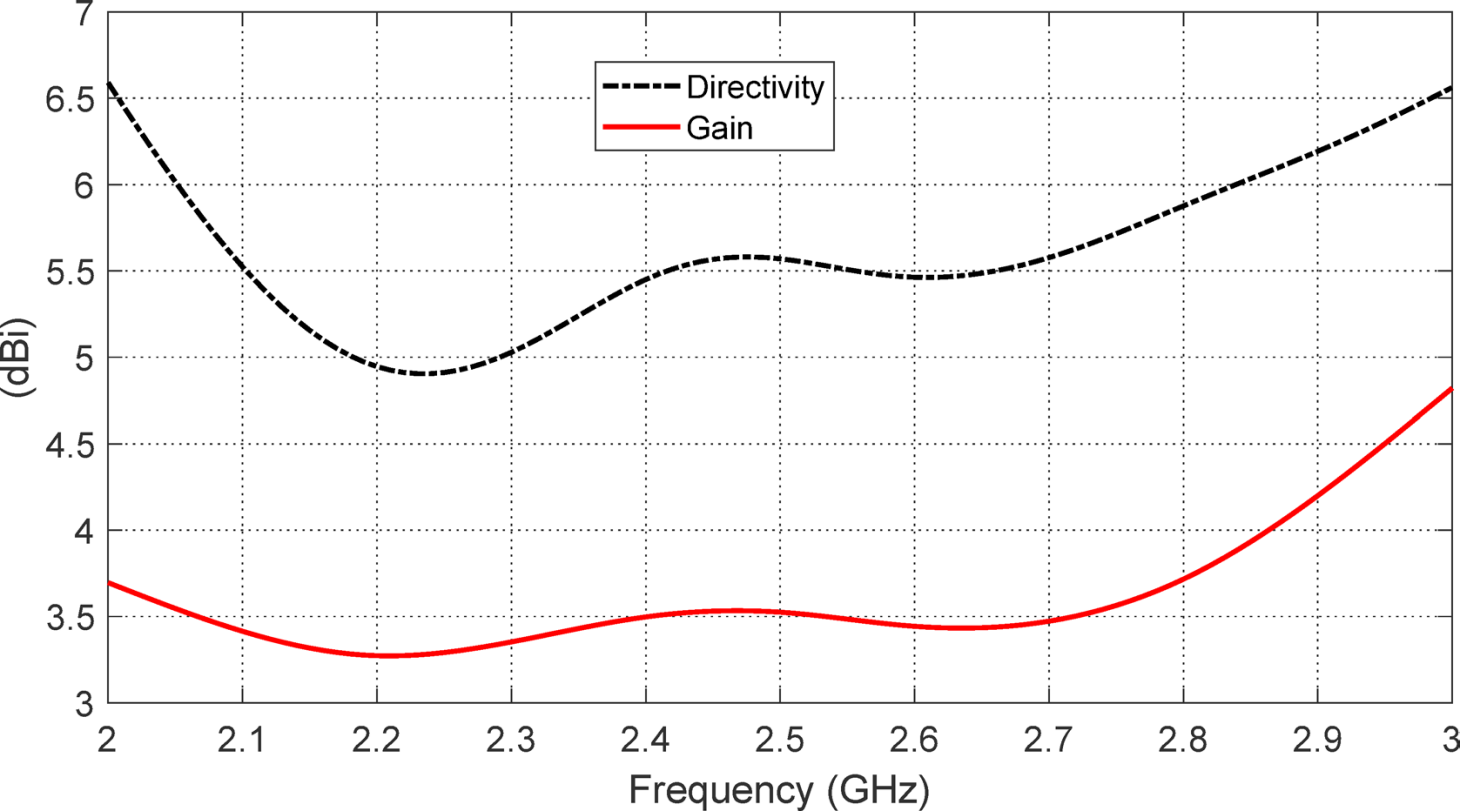
Simulation results for variation in the maximum gain and directivity of the proposed on the stem model over desired frequency band.

The changes in the maximum gain and directivity of the proposed antenna when placed on the stem model over desired frequency band are shown in Fig. 21. The directivity is much higher than the gain. That reflects the low radiation efficiency of the presented antenna. Consequently, the presented antenna is capable of restricting the output power in stem tissues.

#### Antenna efficiency

**Fig. 22:**
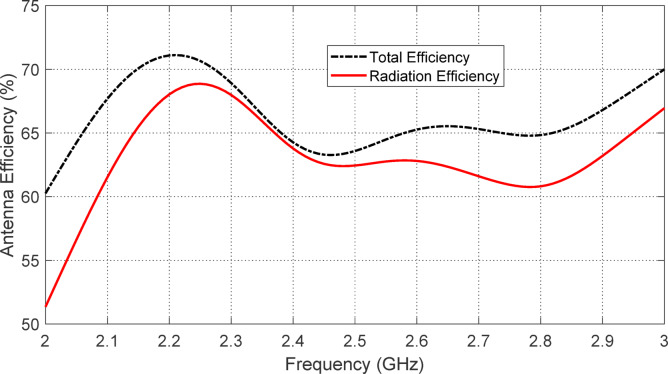
Simulation results for variation in the total and radiation efficiencies of the two arms antennas on the plant stem model over desired frequency band.

The total and radiation efficiencies of the proposed antenna over desired frequency band when placed on a stem model are shown in Fig. 22. This Figure indicates that about 35% of the power radiation penetrates the stem in most of the frequency band. However, this is considered sufficient for detecting internal changes in the stem.

### Selection of frequency range for maximum sensitivity to internal cavities

To construct an effective detection system for cavities inside the plant stem, the mutual S-parameter between the two antennas ($\:{S}_{21}$) should be sensitive to the changes of the cavity diameter ($\:D$). This section presents important simulation results to investigate the sensitivity of the two-element antenna system to the changes of the cavity size in the stem pith.

Mutual coupling between the two antennas indicates the transmission of the wave from the transmitting antenna to the receiving antenna over the medium through which it propagates. It reflects the effect of the medium on wave propagation between the two antennas. Mutual coupling between two antennas is therefore sensitive to the size and location of the cavities embedded inside the stem pith irrespective of their depth below from the stem surface.

**Fig. 23:**
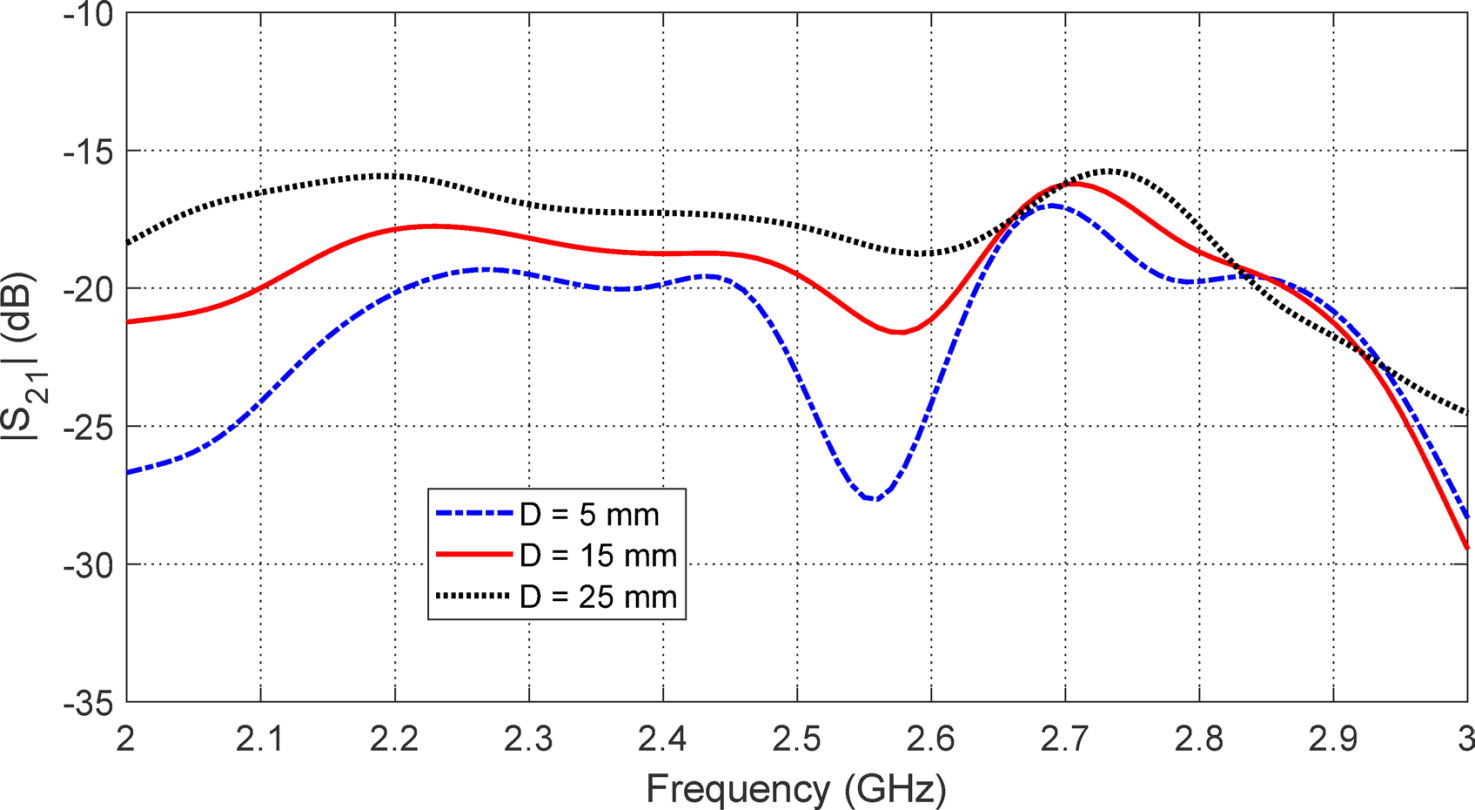
Simulation results for frequency response of $\:\left|{S}_{21}\right|$ for different cavity diameters ($\:D=5,\:15,$ and $\:25\:\mathrm{m}\mathrm{m}$). The cavity location is$\:\:r=25\:\mathrm{m}\mathrm{m}\:$, $\:\:\phi\:=30^\circ\:$, and the antenna clearance is $\:d=2\:\mathrm{m}\mathrm{m}$.

Figure 23 presents the frequency response of $\:\left|{S}_{21}\right|$ for different cavity diameters ($\:D=5,\:15,$ and $\:25\:\mathrm{m}\mathrm{m}$), while the cavity position is fixed at $\:r=25\:\mathrm{m}\mathrm{m}$, $\:\phi\:=30^\circ\:$. The results clearly show that of $\:\left|{S}_{21}\right|$ varies significantly with cavity size.

Larger cavities introduce stronger dielectric discontinuities, resulting in increased scattering and attenuation along the propagation path between the antennas. This leads to more pronounced deviations in the mutual coupling response. These observations confirm that $\:\left|{S}_{21}\right|$ can be effectively utilized to estimate the size of cavities embedded within the stem pith.

The selection of the optimal frequency band for cavity detection is based on the normalized differential sensitivity defined in Sect.  7.2. Specifically, the operating band is chosen to maximize the sensitivity of the mutual coupling parameter to variations in the cavity diameter ($\:D$), as quantified by the normalized differential sensitivity $\:{\widehat{S}}_{D}$ defined in (7).

It should be noted that, in all subsequent simulation results related to the sensitivity analysis, the variations in the cavity diameter ($\:D)$ for the differential sensitivity metric is evaluated using fixed perturbation $\:\varDelta\:D=10\:\mathrm{m}\mathrm{m}$.

**Fig. 24:**
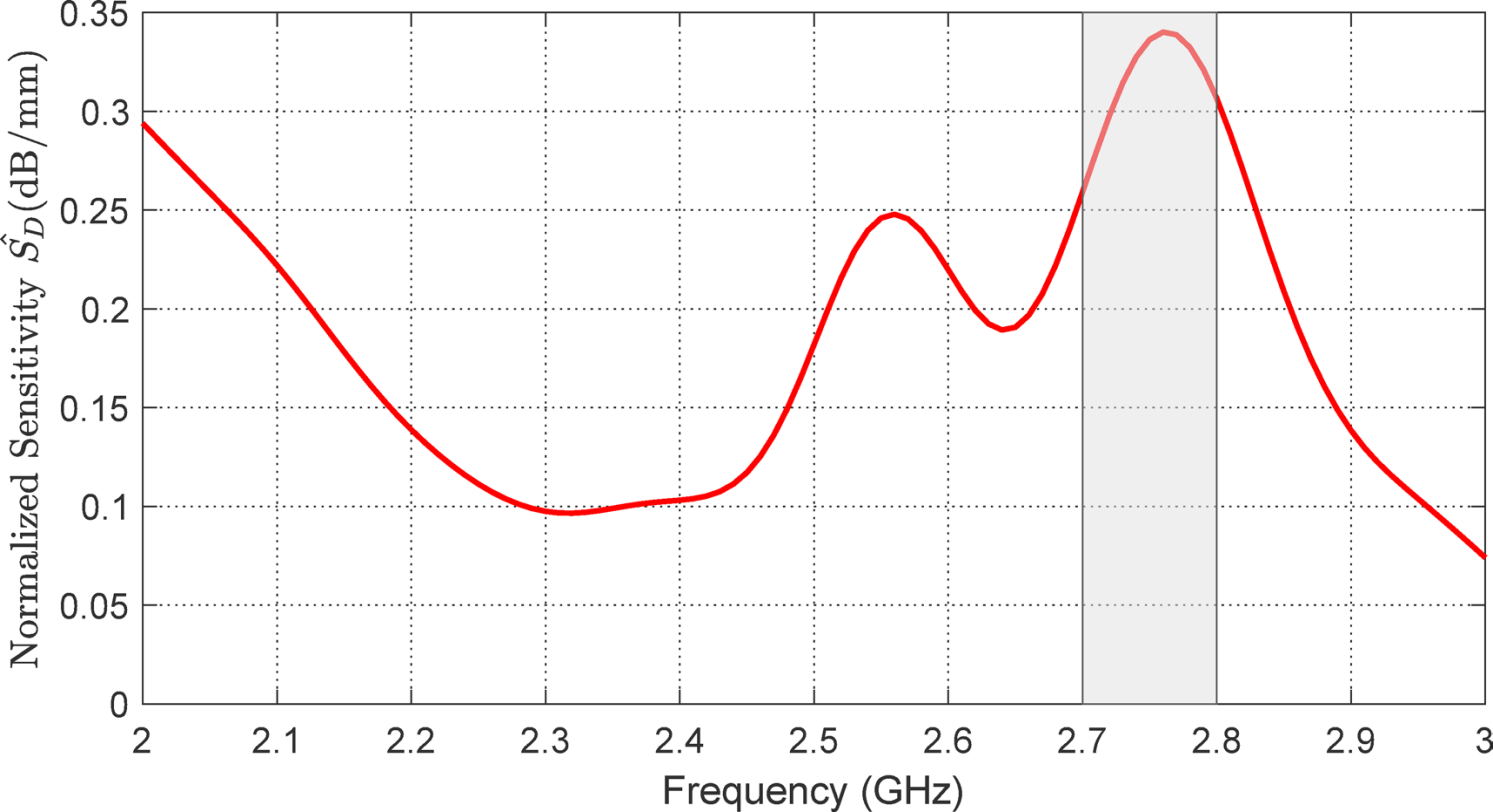
Simulation results for variation of the normalized differential sensitivity ($\:{\widehat{S}}_{D}$) with the frequency for changes in the embedded cavity diameter ($\:D$). Perturbation values is $\:\varDelta\:D=10\:\mathrm{m}\mathrm{m}$.

Figure 24 illustrates the variation of this normalized differential sensitivity metric as a function of frequency while perturbing the embedded cavity diameter. It can be observed that the sensitivity exhibits a frequency-dependent behavior, with distinct regions where the response to changes in cavity diameter is significantly enhanced.

Over the frequency range of $\:2.7\:-\:2.8\:\mathrm{G}\mathrm{H}\mathrm{z}$, the normalized differential sensitivity ($\:{\widehat{S}}_{D}$) ranges between $\:0.25-0.35\:\mathrm{d}\mathrm{B}/\mathrm{m}\mathrm{m}$ and reaches a maximum value at $\:2.76\:\mathrm{G}\mathrm{H}\mathrm{z}$, This value indicate that relatively small variations in the cavity diameter produces clearly measurable changes in the mutual coupling response, confirming the high detection capability of the proposed sensing configuration.

As discussed before in Sect.  4.1 (Fig. 6), both permittivity and conductivity exhibit only weak frequency dependence over the investigated band. Therefore, the optimum sensitivity frequency around 2.76 GHz is not a consequence of strong material dispersion, but rather of the electromagnetic interaction between the antenna near fields and cavity-induced perturbations within the stem.

The presented results confirm that the mutual coupling parameter $\:\left|{S}_{21}\right|$ is highly sensitive to variations in cavity size making it a reliable observable for non-destructive detection of internal stem defects. By analyzing the variations in $\:\left|{S}_{21}\right|$, it is possible to infer the size of embedded cavities.

### Experimental work for measuring the scattering parameters

For antenna performance evaluation, a pair of the proposed planar dipole antenna with proper angular separation is used to form a conceptual microwave radar sensing unit for detecting embedded cavities associated with tree diseases (this is discussed in-detail in Sect.  7). Variations in both the self- and mutual-scattering parameters of this antenna pair are exploited as indicators of the presence, size, and spatial location of internal inhomogeneities within the stem core.

To evaluate the robustness of the proposed antenna with respect to stem geometry, experimental measurements were conducted using stems with diameters of 2 cm, 4 cm, 6 cm, 8 cm, and 10 cm. For each diameter, the scattering parameters were measured repeatedly (ten times), and the reported results correspond to averaged values.

In addition, each measurement configuration was repeated ten times to reduce random measurement variations and improve repeatability. The good agreement between simulation and measurement across this range of stem dimensions confirms the stable performance of the proposed antenna under practical geometric variations.

### Measurement of antenna self S-parameter

A vector network analyzer (VNA) is employed to measure the scattering parameters of the proposed antenna system in its minimum configuration, consisting of two identical antennas arranged with an angular separation of $\:{{\Phi\:}}_{A}=90^\circ\:$ around the plant stem.

Prior to measurement, the VNA was calibrated using a standard SOLT (Short–Open–Load–Through) calibration procedure over the frequency range 2–3 GHz. The calibration reference planes were established at the coaxial cable terminals connected to the antenna ports.

To evaluate measurement repeatability, each experimental configuration was measured ten times after removing and repositioning the antennas on the stem while maintaining the prescribed clearance and angular separation. The scattering parameters presented throughout this paper correspond to the average values obtained from these repeated measurements.

**Fig. 25:**
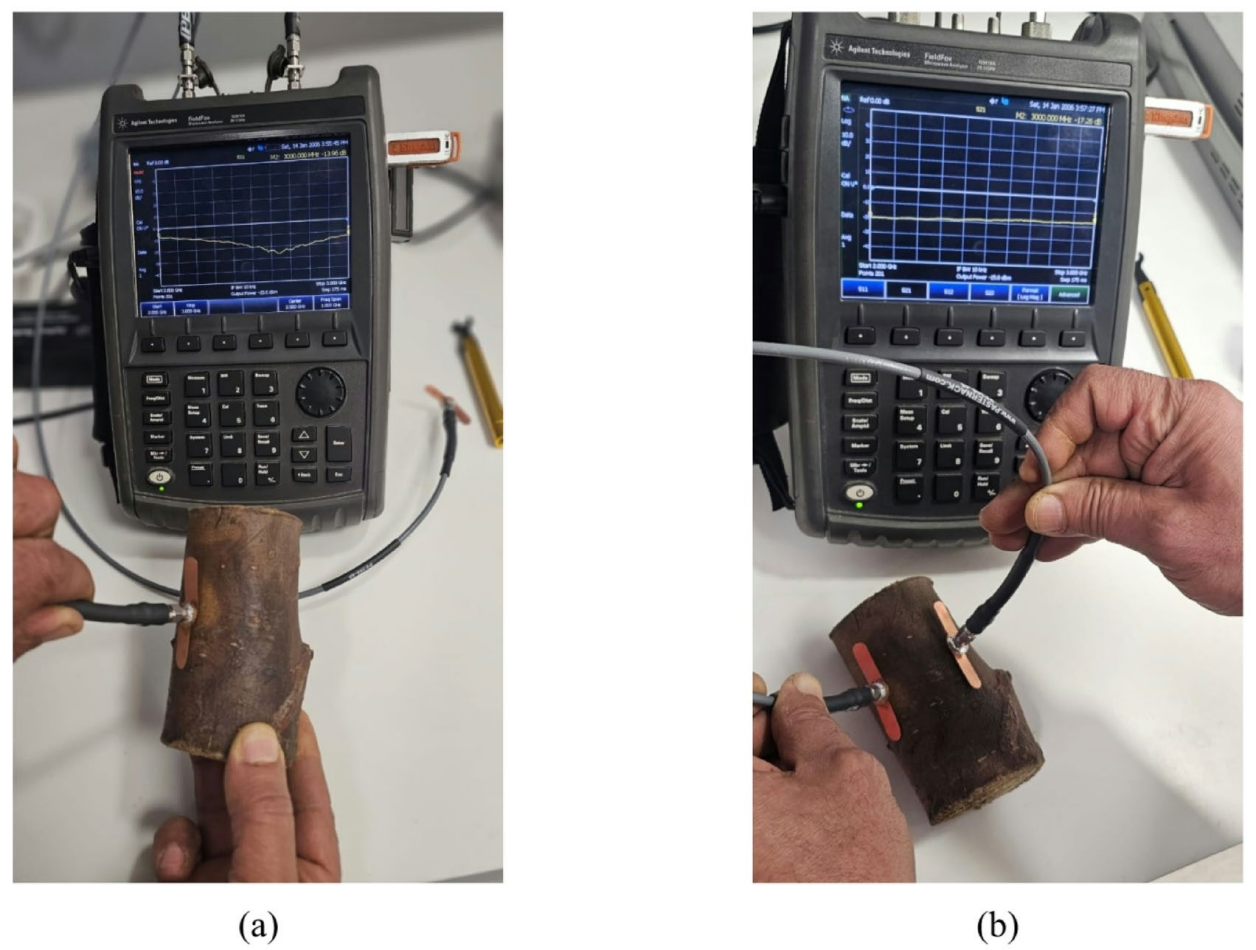
illustrates the experimental measurement setup. In Fig. 25 (a), the self-scattering parameter $\:{S}_{11}$ is measured for a single antenna placed adjacent to the plant stem, with the dipole arms oriented parallel to the stem axis and separated from the surface by a clearance of approximately $\:2\:\mathrm{m}\mathrm{m}$. In Fig. 25 (b), two antennas are mounted on the stem with an angular separation of $\:{{\Phi\:}}_{A}=90^\circ\:$, and the full set of scattering parameters ($\:{S}_{11}$, $\:{S}_{22}$, and $\:{S}_{21}$) is measured.

Figure 25: VNA–based measurement configuration for experimental validation of the proposed microwave sensing antenna: (**a**) Single-antenna setup for evaluating impedance matching ($\:{S}_{11}$) in close proximity to the stem; (**b**) Dual-antenna configuration with an angular separation of $\:{{\Phi\:}}_{A}=90^\circ\:$ used to measure self- and mutual-scattering parameters ($\:{S}_{11}$, $\:{S}_{22}$, and $\:{S}_{21}$) for internal inhomogeneity detection. Averaged measurements are obtained for stems with diameters ranging from 2 cm to 10 cm. Each measurement configuration was repeated ten times.

**Fig. 26:**
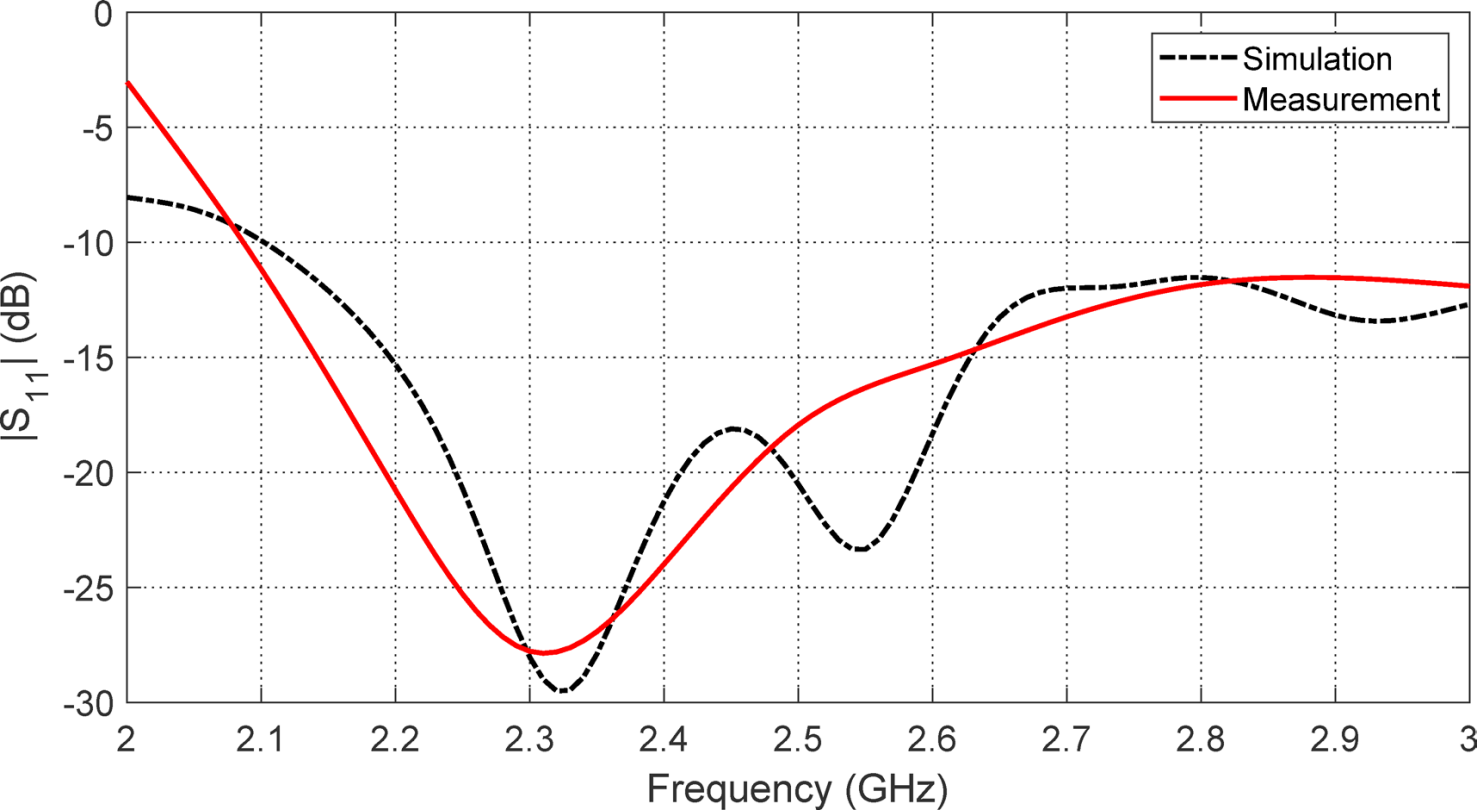
Simulation and measurement results showing the variation of the magnitude of self-scattering parameter, $\:\left|{S}_{11}\right|$, with the frequency over the range $\:2-3\:\mathrm{G}\mathrm{H}\mathrm{z}$. Averaged measurements are obtained for stems with diameters ranging from 2 cm to 10 cm. Each measurement configuration was repeated ten times.

**Fig. 27:**
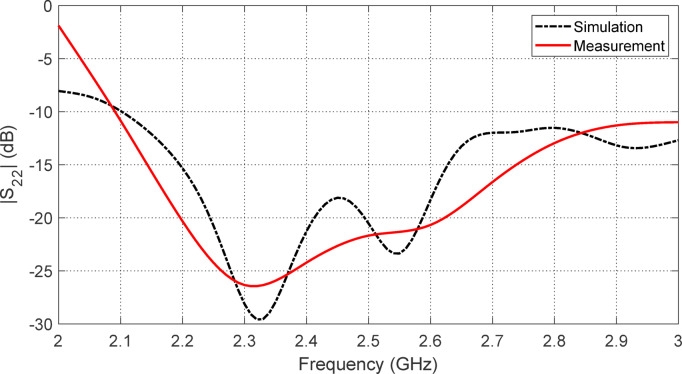
Simulation and measurement results showing the variation of the magnitude of self-scattering parameter, $\:\left|{S}_{22}\right|$, with the frequency over the range $\:2-3$ GHz. Averaged measurements are obtained for stems with diameters ranging from 2 cm to 10 cm. Each measurement configuration was repeated ten times.

The variations of the magnitudes of the self-scattering parameters $\:\left|{S}_{11}\right|$ and $\:\left|{S}_{22}\right|$ over the frequency range of $2 - 3\:G{\mathrm{Hz}}$ as obtained by simulation and measurement are presented in Figs. 26 and 27, respectively. It is observed that both $\:\left|{S}_{11}\right|$ and $\:\left|{S}_{22}\right|$ remain below $\:-10\:\mathrm{d}\mathrm{B}\:$over a wide frequency band extending from approximately $\:2.1\:\mathrm{G}\mathrm{H}\mathrm{z}\:$to beyond $\:3\:\mathrm{G}\mathrm{H}\mathrm{z}$. This confirms that the proposed antenna exhibits good impedance matching across the operating band, which is sufficiently wide to support reliable detection of cavities and decay regions within the stem.

### Measurement of the mutual S-parameters for antenna pair

**Fig. 28:**
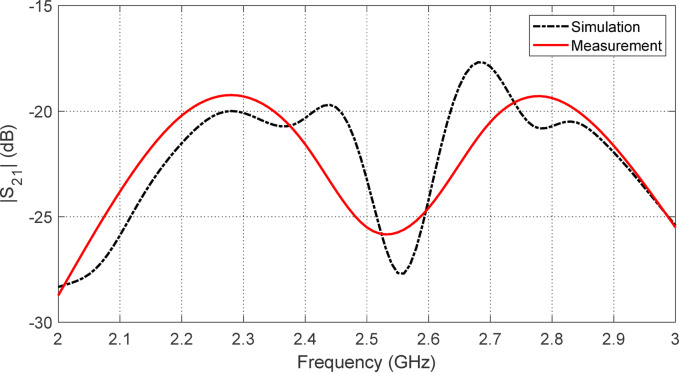
Simulation and experimental results for the variation of the magnitude of the mutual S-parameter $\:\left|{S}_{21}\right|$ for the two antennas placed on a healthy stem (free from cavities and decays) over the frequency range $\:2-3\:\mathrm{G}\mathrm{H}\mathrm{z}$. The angular separation between the two antennas is $\:{\phi\:}_{A}=90^\circ\:$. The clearance of the antennas above the stem surface is $\:h=2\:\mathrm{m}\mathrm{m}$.

The mutual coupling between the two antennas is evaluated through the magnitude of the mutual scattering parameter $\:\left|{S}_{21}\right|$. As shown in Fig. 28, both simulation and measurement results indicate that $\:\left|{S}_{21}\right|$ remains below − 18 dB when the antennas are placed on a healthy stem free from cavities and decay. This means that such a couple of antennas are capable of detecting the embedded cavities or decays by measuring the change of the $\:\left|{S}_{21}\right|$ due to the existence of such inhomogeneities inside the stem. This is performed through sensitivity analysis, as explained in Sect.  7.2.

Any deviation from this baseline, caused by internal cavities or decay regions, is expected to produce a measurable change in $\:\left|{S}_{21}\right|$. Consequently, the proposed antenna pair demonstrates strong potential for detecting internal inhomogeneities through mutual-coupling-based sensing. A detailed sensitivity analysis addressing these effects is presented in a subsequent section.

### Comparison with published related work

Table 5(a) and 5(b) provide a comprehensive comparison between the proposed system and representative microwave-based techniques for detecting internal cavities in wood and plant structures. The tables clearly reveal both methodological and performance-level differences. As shown in Table 5(a), most existing approaches, such as^15–17^ and ^18^, are based on microwave tomography or time-reversal frameworks, typically operating with multi-antenna or multi-static configurations.

These systems demonstrate varying levels of cavity detection capability. The proposed work is capable of detecting $\:4\:\mathrm{m}\mathrm{m}$ cavity in a $\:10\mathrm{c}\mathrm{m}$ trunk (4% of trunk diameter). In comparison^15^, detected a $\:5.5\:\mathrm{m}\mathrm{m}$ cavity inside a $\:30\:\mathrm{c}\mathrm{m}$ trunk, corresponding to approximately $\:1.8\%$ of the trunk diameter, however its performance is constrained by diffraction limits, model inaccuracies, and the high computational burden associated with solving ill-posed inverse problems. Similarly^16^, , detected cavities of 6 cm in a 15 cm trunk, corresponding to $\:33\%$ of the trunk diameter, but required a dense multi-static antenna array, increasing hardware complexity and cost^17^., detected 6 cm cavities inside a $\:46\:\mathrm{c}\mathrm{m}$ trunk, representing nearly $\:13\%$ of the trunk diameter, although the system relied on a large number of sensors. Likewise^18^ employed a multi-antenna microwave tomography system capable of detecting $\:5\:\mathrm{c}\mathrm{m}$ cavities in a 32 cm trunk, corresponding to approximately $\:15.6\%$ of the trunk diameter, nevertheless, the setup was implemented as a bulky laboratory-scale system, limiting portability and practical field deployment. For^19^ and ^20^, the minimum detectable cavity size relative to trunk diameter was not reported.

Ground-penetrating radar (GPR)-based approaches, including^21,22^ also exhibit important limitations. In^22^, cavities of 6 cm were detected in a 19.5 cm trunk, corresponding to approximately 31% of the trunk diameter, however, the system required bulky VNA equipment and showed limited capability for detecting small natural cavities. Similarly^21^, detected 6 cm cavities in a 30 cm trunk (20% of trunk diameter), but, the validation was limited to a single tree scenario and required complex antenna fabrication.

The proposed system operates within a comparable 2–3 GHz band The smallest perturbation investigated in this work corresponds to $\:{\Delta\:}D=4\:\mathrm{m}\mathrm{m}$ owing to its reliance on near-field electromagnetic interaction rather than far-field imaging, enabling sensitivity beyond the conventional diffraction limit. Consequently, the proposed approach demonstrates significantly improved sensitivity compared with most previous experimental studies and achieved using a much simpler sensing configuration without complex tomography or inverse reconstruction algorithms.

Since measurable changes in |$\:{S}_{21}$| were observed for this perturbation ($\:{\Delta\:}D=4\:\mathrm{m}\mathrm{m}$), the experimental evidence supports sensitivity to cavity size changes on the order of a few millimeters. Nevertheless, this should not be interpreted as a demonstrated imaging resolution limit.

Table 5(b) further emphasizes the practical advantages of the proposed approach. None of the referenced works explicitly address medium adaptivity (with the exception of^20^, which did not provide cavity-detection validation), as most existing methods assume fixed or homogeneous dielectric properties, which can lead to performance degradation under varying moisture content or biological variability. The proposed system, however, maintains stable operation across a range of dielectric conditions, providing a distinct advantage for realistic plant stem environments. Additionally, existing tomography-based systems are characterized by high or very high system complexity, requiring multiple antennas, calibration procedures, and iterative reconstruction algorithms, which limit portability and real-time applicability. Even simpler systems, such as GPR, only achieve moderate reductions in complexity. In contrast, the proposed two-element near-field antenna system.

significantly reduces hardware and computational requirements by eliminating the need for inverse reconstruction, resulting in a low-complexity and portable solution. Although the proposed method does not aim to produce full 3D images, it instead offers a direct, high-sensitivity detection mechanism for internal cavities, which is more suitable for rapid, in-situ diagnostics. Overall, the tables demonstrate that the contribution of this work lies not merely in incremental performance improvement, but in introducing a fundamentally different sensing paradigm that achieves superior sensitivity, robustness, and practicality within a compact and efficient system design.

**Table 5a:** Quantitative comparison between the proposed antenna system and representative state-of-the-art microwave-based techniques for detecting internal cavities in wood and plant structures (system and performance metrics).

Work	Antenna System	Technique	Frequency Band (GHz)	Minimum detectable size $\:\left(\boldsymbol{D}/{\boldsymbol{D}}_{\boldsymbol{T}}\right)$
^15^	UWB antenna array	Microwave imaging + inverse problem	0.9–20 GHz	$\:5.5\mathrm{m}\mathrm{m}/30\mathrm{c}\mathrm{m}$
^16^	Multi-static array	Time-reversal tomography	1–5 GHz	$\:5\mathrm{c}\mathrm{m}/15\mathrm{c}\mathrm{m}$
^17^	Sensor array	Microwave tomography	1 GHz	$\:6\mathrm{c}\mathrm{m}/46\mathrm{c}\mathrm{m}\:$ (weak detection)
^18^	Multi-antenna	Microwave tomography	0.4–1.4 GHz	$\:5\mathrm{c}\mathrm{m}\:/\:32\mathrm{c}\mathrm{m}$
^19^	GPR antennas	GPR + microwave tomography	0.8–1.8 GHz Simulation 1.2–2.3 GHz	$\:\mathrm{N}\mathrm{A}$
^20^	Single antenna	Microwave Imaging	UWB	$\:\mathrm{N}\mathrm{A}$
^21^	Vivaldi, dual polarization	GPR- based microwave imaging	0.5–3 GHz	$\:6\mathrm{c}\mathrm{m}/30\mathrm{c}\mathrm{m}$
^22^	Rader vector network analyzer	VNA Radar + Radon transform	0.3–8 GHz	$\:6\mathrm{c}\mathrm{m}\:/19.5\mathrm{c}\mathrm{m}$
Proposed	Two-Element Antenna System	Near Field Sensing	2–3 GHz	$\:4\mathrm{m}\mathrm{m}\:/10\mathrm{c}\mathrm{m}$

**Table 5b:** Quantitative comparison between the proposed antenna system and representative state-of-the-art microwave-based techniques for detecting internal cavities in wood and plant structures (practical and design aspects).

Work	Medium-Adaptivity	Portability	System Complexity	Key Limitations
[15]	$\:\times\:$	$\:\times\:$	High (inverse solver)	Computationally expensive, ill-posed
[16]	$\:\times\:$	$\:\times\:$	Very High	Requires dense antenna array
[17]	$\:\times\:$	$\:\times\:$	High	Large number of sensors required
[18]	$\:\times\:$	$\:\times\:$	High	Bulky, lab-scale setup
[19]	$\:\times\:$	$\checkmark$	Moderate	Limited resolution, weak small-defect sensitivity
[20]	$\checkmark$	$\checkmark$	Low	No imaging validation
[21]	$\:\times\:$	$\:\times\:$	High	Limited validation tested on only one tree scenario. Complex fabrication.
[22]	$\:\times\:$	$\checkmark$	Moderate	Limited detection capability for small natural cavities (~ 2 cm), requires bulky VNA setup
Proposed	$\checkmark$	$\checkmark$	Low	Eliminates inverse problem, high sensitivity to small cavities.

## Discussion, limitations, and future work

The principal contribution of this work is the development of a medium-adaptive wideband near-field antenna whose performance is optimized for operation adjacent to a lossy, anisotropic, and dispersive plant stem.

Unlike conventional wideband dipoles that are primarily designed for free-space radiation, the proposed antenna maintains broadband impedance matching under strong dielectric loading, provides balanced current excitation through a dual-toroidal-ring balun, and exhibits quantitatively verified sensitivity to internal cavities through both self- and mutual-scattering parameters.

The sensing capability is evaluated using dedicated differential and overall sensitivity metrics that relate variations in the mutual coupling response to changes in cavity size and location, thereby linking antenna performance directly to defect-detection capability.

Future work will include quantitative characterization of the proposed balun using arm-current imbalance metrics and common-mode current measurements, together with comparative studies against conventional sleeve- and choke-balun implementations under identical stem-loading conditions.

It should be emphasized that the present work focuses on the antenna and sensing aspects of the proposed microwave inspection approach. The objective is to establish that the scattering parameters of the antenna system exhibit sufficient sensitivity to internal cavities. Performance metrics such as detection accuracy, false-positive rate, false-negative rate, ROC curves, and classification thresholds require the development of a complete detection and decision-making framework based on extensive experimental datasets. The development and validation of such a framework constitute the subject of future work.

It should be noted that real plant stems may contain various naturally occurring dielectric inhomogeneities, such as knots, cracks, moisture pockets, and anatomical variations. While such features can also influence the measured scattering parameters, the objective of the present work is to establish the sensitivity of the proposed antenna system to internal dielectric discontinuities in general and to cavity-type defects in particular.

Reliable discrimination between different defect types is expected to require a larger multi-antenna configuration combined with advanced signal-processing or machine-learning algorithms that exploit the spatial and spectral signatures of the measured mutual coupling data. The development of such classification and localization techniques constitutes an important direction for future research.

The proposed two-element configuration should, therefore, be viewed as a feasibility-study platform for antenna evaluation and sensitivity assessment, whereas practical field deployment is expected to employ a larger antenna array providing improved localization capability and reduced false-alarm probability. While the compact dimensions of the proposed antenna enable the deployment of multiple sensing elements around the stem circumference, mutual coupling between neighboring antennas becomes an important consideration in dense array configurations.

Additional coupling paths may alter the measured scattering parameters and complicate the interpretation of cavity-induced responses. Potential mitigation strategies include optimized angular spacing, calibration-based compensation, passive decoupling techniques, and multiport signal-processing algorithms. Future work will investigate four-element and larger sensing configurations to evaluate these effects and develop robust cavity-localization methodologies.

The proposed sensing approach is based on near-field electromagnetic coupling between antennas mounted directly on the stem surface. Unlike conventional far-field radar systems, most of the transmitted energy is confined within the stem tissues, reducing sensitivity to distant objects and external electromagnetic interference. Although nearby vegetation and environmental structures may introduce secondary perturbations to the measured scattering parameters, their influence is expected to be considerably smaller than that of internal dielectric discontinuities located within the dominant coupling region.

Additional robustness can be achieved through baseline calibration, frequency averaging, and multi-antenna measurements. Experimental evaluation of the system under realistic outdoor conditions will be considered in future work. The present investigation is limited to a single-cavity model in order to establish the fundamental sensing characteristics of the proposed antenna system. In practical stems, multiple cavities, cracks, decay regions, or irregularly shaped defects may coexist.

Such inhomogeneities would collectively contribute to the measured scattering response through the superposition of their individual scattered fields. While the proposed antenna is expected to remain sensitive to these defects due to their dielectric contrast with the surrounding wood tissues, accurate characterization of multiple defects would require additional measurement diversity.

Future work will therefore focus on extending the sensing architecture to multi-antenna configurations combined with advanced signal-processing algorithms for localization and characterization of multiple and irregularly shaped defects. It may be worth noting that the experimental work presented in this work is intended to validate the electromagnetic performance of the proposed antenna system, including impedance matching and mutual coupling characteristics under realistic mounting conditions.

Experimental validation of cavity detection using stems containing artificial defects of known dimensions and locations is beyond the scope of the present study and will be addressed in future work. Such experiments will enable quantitative determination of detection limits, minimum detectable cavity size, and robustness against measurement uncertainties.

An additional advantage of the proposed methodology is its low computational complexity. Since cavity detection is based on direct analysis of measured scattering parameters and the evaluation of sensitivity metrics, the required computations scale approximately as $\:O\left({N}_{f}\right)$, where $\:{N}_{f}$ is the number of measured frequency samples. This is substantially less demanding than microwave tomography techniques, which require iterative solutions of forward and inverse electromagnetic problems for image reconstruction. As a result, the proposed approach is well suited for rapid and low-cost field deployment.

## Conclusion

This paper has presented a medium-adaptive wideband near-field antenna for non-destructive detection of internal cavities in plant stems. Unlike conventional antenna designs optimized for free-space operation, the proposed structure is specifically engineered to operate in close proximity to a lossy and heterogeneous biological medium. The integration of a compact quasi-planar dipole with a dual-ring balun enables balanced current excitation and stable impedance matching over the 2.0–3.0 GHz frequency band, even under strong dielectric loading conditions.

A conceptual two-element antenna configuration has been introduced as a feasibility study to evaluate the capability of the proposed antenna for cavity detection. A sensitivity-driven framework has been developed to quantify the response of the system to variations in cavity diameter, radial depth, and angular position. The results demonstrate that the mutual coupling parameter $\:\left|{S}_{21}\right|$ provides a reliable and informative observable for detecting and characterizing internal defects, while the reflection coefficient offers complementary sensitivity to near-surface inhomogeneities.

Based on the sensitivity analysis, optimal operating frequency band centered at $\:2.76\:\mathrm{G}\mathrm{H}\mathrm{z}$ and appropriate antenna clearance have been identified to maximize detection performance. The proposed design and sensing methodology have been validated through both full-wave simulations and experimental measurements, confirming the consistency of the results and the robustness of the approach.

The compact and planar geometry of the antenna enables straightforward integration into multi-element configurations, offering a scalable framework for improved localization and characterization of internal cavities. Future work will focus on the development of multi-antenna systems and advanced signal processing algorithms for accurate reconstruction of cavity parameters in real-world forestry and agricultural environments.

## Data Availability

The datasets used and/or analyzed during the current study available from the corresponding author on reasonable request.
